# SRC kinase drives multidrug resistance induced by KRAS-G12C inhibition

**DOI:** 10.1126/sciadv.adq4274

**Published:** 2024-12-11

**Authors:** Xinxin Song, Zhuan Zhou, Ammar Elmezayen, Runliu Wu, Chunhua Yu, Boning Gao, John D. Minna, Kenneth D. Westover, Herbert J. Zeh, Guido Kroemer, Lynn E. Heasley, Rui Kang, Daolin Tang

**Affiliations:** ^1^ Department of Surgery, UT Southwestern Medical Center, Dallas, TX 75390, USA.; ^2^ Departments of Biochemistry and Radiation Oncology, UT Southwestern Medical Center, Dallas, TX 75390, USA.; ^3^ Hamon Center for Therapeutic Oncology Research, Department of Pharmacology, Department of Internal Medicine, Harold C. Simmons Comprehensive Cancer Center, UT Southwestern Medical Center, Dallas, TX 75390, USA.; ^4^ Centre de Recherche des Cordeliers, Equipe labellisée par la Ligue contre le cancer, Université de Paris, Sorbonne Université, INSERM U1138, Institut Universitaire de France, Paris, France.; ^5^ Metabolomics and Cell Biology Platforms, Gustave Roussy Cancer Campus, Villejuif, France.; ^6^ Pôle de Biologie, Hôpital Européen Georges Pompidou, AP-HP, Paris, France.; ^7^ Department of Craniofacial Biology, University of Colorado Anschutz Medical Campus, Aurora, CO 80045, USA.; ^8^ Eastern Colorado VA Healthcare System, Rocky Mountain Regional VA Medical Center, Aurora, CO 80045, USA.

## Abstract

Direct targeting of the *KRAS-G12C*–mutant protein using covalent inhibitors (G12Ci) acts on human non–small cell lung cancer (NSCLC). However, drug resistance is an emerging concern in this approach. Here, we show that MRTX849, a covalent inhibitor targeting the *KRAS-G12C* mutation, leads to the reactivation of the mitogen-activated protein kinase signaling pathway in MRTX849-resistant NSCLC and pancreatic ductal adenocarcinoma. A genome-wide CRISPR screen revealed that the adenosine triphosphate binding cassette transporter ABCC1 mediates MRTX849 resistance. Functional studies demonstrated that the transcription factor JUN drives ABCC1 expression, resulting in multidrug resistance. An unbiased drug screen identified the tyrosine kinase inhibitor dasatinib that potentiates MRTX849 efficacy by inhibiting SRC-dependent JUN activation, avoiding multidrug resistance and tumor suppression in vitro as well as in suitable preclinical mouse models and patient-derived organoids. SRC inhibitors (DGY-06-116, dasatinib, and bosutinib) also exhibit synergistic effects with MRTX849 in eliminating various tumor cell lines carrying *KRAS-G12C* mutations. Thus, SRC inhibitors amplify the therapeutic utility of G12Ci.

## Teaser

Targeting SRC overcomes G12Ci resistance in *KRAS-G12C* mutant cancers.

## INTRODUCTION

Normally, the KRAS protein switches between the inactive form of guanosine diphosphate (GDP)–RAS and the active form of guanosine triphosphate (GTP)–RAS (*1*). However, the *KRAS* gene is frequently mutated in a variety of human cancers, especially non–small cell lung cancer (NSCLC), colorectal cancer, and pancreatic ductal adenocarcinoma (PDAC), resulting in the oncogenic activation of the KRAS protein (*2*, *3*). The KRAS protein was long considered to be “undruggable” until the identification of molecules that target the *KRAS-G12C* (glycine-12 to cysteine) mutant in a covalent manner (*4*–*8*). Certain covalent KRAS-G12C inhibitors (G12Ci), including AMG510 (*9*, *10*) and MRTX849 (*11*, *12*), have been introduced into clinical trials. Specifically, AMG510 (also known as sotorasib) is the first G12Ci approved by the US Food and Drug Administration (FDA) in 2021 for the treatment of adult patients with *KRAS-G12C*–mutated NSCLC. MRTX849 (also known as adagrasib) is more selective for KRAS-G12C protein, showing a 42.9% overall response rate and a disease control rate of 80% in a phase 2 trial (*12*). Although this is an important milestone in precision oncology, G12Ci treatments are facing challenges due to the emerging development of drug resistance (*13*, *14*).

Preclinical and clinical studies have elucidated the diverse mechanisms underlying resistance to G12Ci (*15*). These include the activation of additional oncogenes [e.g., *NRAS*, b-Raf proto-oncogene (*BRAF*), mitogen-activated protein kinase 1 (*MAP2K1*/*MEK1*), *ALK*, *MET*, and *RET*] by point mutation or fusion events; the inactivation of the tumor suppressor phosphatase and tensin homolog (*PTEN*) (*13*, *16*); impaired KRAS nucleotide cycling (*17*); abnormal KRAS vertical signaling (*18*); wild-type (WT) RAS (NRAS and HRAS) activation (*19*); the activation of receptor tyrosine kinases [e.g., epidermal growth factor receptor (EGFR), erbB2/3 receptor tyrosine kinase (ERBB2/3), fibroblast growth factor receptor 1 (FGFR1), or AXL] (*20*, *21*); the Yes-associated protein (YAP) pathway (*22*); cell cycle dysregulation (*23*); epithelial-to-mesenchymal transition (*24*); and perturbation of proteostasis (*25*, *26*). However, how long-term use of G12Ci leads to multidrug resistance, a phenomenon in which cancer cells exposed to one anticancer drug exhibit resistance to various additional drugs, remains poorly understood (*27*). Understanding the mechanisms and drivers of multidrug resistance is critical for predicting the sensitivity of G12Ci therapies or the benefit of their clinical applications.

In this study, we used next-generation sequencing technologies coupled with genome-wide CRISPR analysis to demonstrate that MRTX849 induces multidrug resistance in human NSCLC and PDAC cells by selectively up-regulating the expression of adenosine triphosphate (ATP) binding cassette subfamily C member 1 (ABCC1) transporter. Our unbiased drug screen identified that the tyrosine kinase inhibitor dasatinib enhanced the efficacy of MRTX849 by inhibiting the SRC-mediated activation of the transcription factor JUN and subsequent ABCC1 expression. Last, we provide proof-of-concept results demonstrating that the pharmacological inhibition of SRC holds great potential as a strategy to eliminate drug-resistant cells during G12Ci therapy.

## RESULTS

### Reactivation of the MAPK signaling pathway in MRTX849-resistant KRAS-G12C–mutant cells

**Fig. 1.**
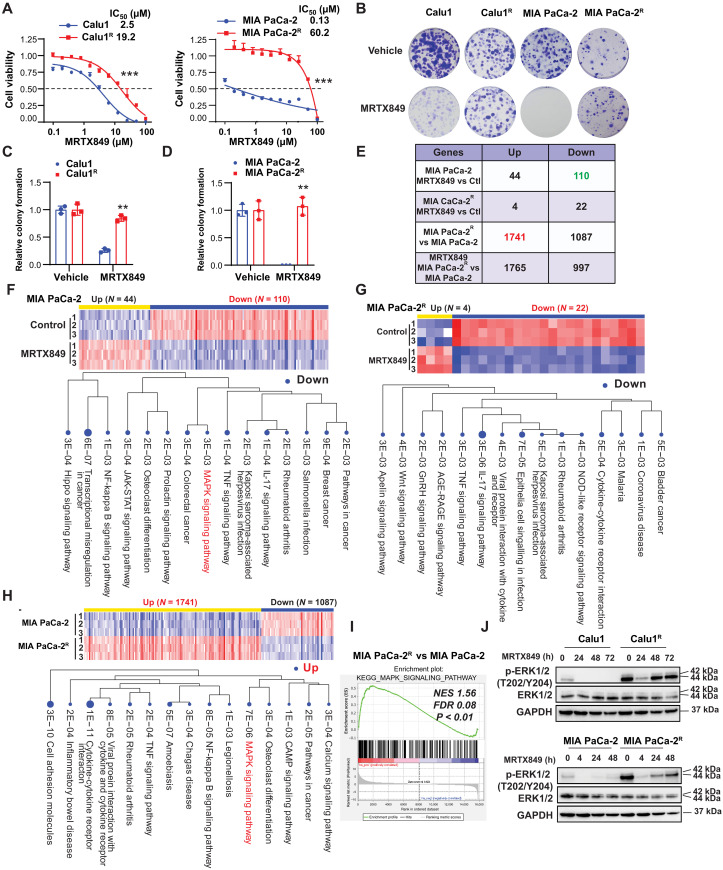
Reactivation of the MAPK signaling pathway in MRTX849-resistant *KRAS-G12C*–mutant cells. (**A**) Calu1 and its KRAS-G12Ci–resistant cells (Calu1^R^) and MIA PaCa-2 and its resistant cells (MIA PaCa-2^R^) were treated with MRTX849 at the indicated concentrations. The percentage of viable cells is shown relative to untreated controls by CCK-8 assay (*n* = 3 biologically independent samples; ***P < 0.001; two-way ANOVA; data are presented as means ± SD). (**B** to **D**) Cell clonogenic assay. The cells were treated with MRTX849 (5 μM) for 24 hours and observed for 7 to 14 days. The cell colonies were stained with 0.1% crystal violet for evaluation. Quantification was performed by ImageJ (*n* = 3 biologically independent samples; **P < 0.01; Student’s *t* test; data are presented as means ± SD). (**E**) Number of differentially expressed genes identified by iDEP96 using an FDR cutoff of 0.1 and a minimum fold change of 2 between the indicated groups. (**F**) KEGG pathway analysis of differentially down-regulated genes in MIA PaCa-2 cells treated with MRTX849 compared to the control. (**G**) KEGG pathway analysis of differentially down-regulated genes in MIA PaCa-2–resistant cells (MIA PaCa-2^R^) treated with MRTX849 compared to the control. (**H**) KEGG pathway analysis of differentially up-regulated genes in MIA PaCa-2–resistant cells (MIA PaCa-2^R^) compared to MIA PaCa-2 cells. (**I**) GSEA showing that the MAPK signaling pathway was statistically up-regulated between MIA PaCa-2^R^ and MIA PaCa-2 cells (nominal *P* value of <0.01). (**J**) Immunoblot analysis of the indicated proteins of cells treated with 1 μM MRTX849 at the indicated time. The blots shown are representative of three repeats. h, hours; KEGG, Kyoto Encyclopedia of Genes and Genomes; GAPDH, glyceraldehyde-3-phosphate dehydrogenase.

In clinical trials, MRTX849 has been administered at doses ranging from 150 daily to 1200 mg daily, according to data presented at the 2019 American Association for Cancer Research on Molecular Targets and Cancer Therapeutics. The total *C*_ave_ at steady state at 600-mg MRTX849 twice daily (human) is 4362 nM (*28*). Thus, the estimated plasma concentration of MRTX849 could range from 500 to 5000 nM. To generate G12Ci-resistant cell lines, we cultured *KRAS-G12C*–mutant cells, including Calu1 (a human NSCLC cell line) and MIA PaCa-2 (a human PDAC cell line), with increasing concentrations of G12Ci MRTX849 (100 nM-50,000 nM) for 6 months (*29*) and we maintained the resistant cell lines with the clinical relevant dose of MRTX849 (1000 nM). Multiple clones were isolated, expanded, pooled, and tested for sensitivity to MRTX849. Using this approach, we obtained anti-G12Ci variants of Calu1 or MIA PaCa-2 cells (referred to as Calu1^R^ or MIA PaCa-2^R^, respectively) that remained viable upon continuous exposure to high MRTX849 concentrations. In response to MRTX849, MIA PaCa-2^R^ and Calu1^R^ cells exhibited median inhibitory concentration (IC_50_) values that were superior to those of parental cells (the IC_50_ for MIA PaCa-2^R^ = 60.2 μM versus MIA PaCa-2 = 0.13 μM; IC_50_ for Calu1^R^ = 19.2 μM versus Calu1 = 2.5 μM; Fig. 1A). Colony formation assays show that MRTX849 is ineffective at reducing colony formation in the resistant cells (Fig. 1, B to D). Notably, G12Ci-resistant cell lines exhibited lower colony formation rates compared to parental lines (Fig. 1B). This may result from selection pressure by anticancer agents, leading to a subset of cells with slower growth rates but enhanced survival mechanisms (*30*). To explore genetic variation associated with G12Ci-induced resistance, we used high-throughput next-generation sequencing to compare transcriptomics between parental and MRTX849-resistant MIA PaCa-2 cells that were cultured in the absence or presence of MRTX849 for 4 hours, followed by principal components analysis to corroborate grouping among replicates, identify biological outliers, and reduce the number of gene “dimensions” to a minimal set of linearly transformed dimensions including PC1 and PC2 (*31*). Principal components analysis of parental versus MRTX849-resistant MIA PaCa-2 and MIA PaCa-2^R^ cells revealed (i) fundamental differences in gene expression between MIA PaCa-2 and MIA PaCa-2^R^ cells in PC1 in the absence of MRTX849 exposure in PC1 and (ii) transcriptional shifts between vehicle and MRTX849-treated cells in PC2 that were attenuated in MIA PaCa-2^R^ cells compared to parental cells (fig. S1A). Next, we conducted an analysis to identify differentially expressed genes [with a false discovery rate (FDR) cutoff of 0.1 and a minimum fold change of 2] that were up-regulated or down-regulated in MIA PaCa-2^R^ cells following exposure to MRTX849 compared to MIA PaCa-2 cells (Fig. 1E). In MIA PaCa-2 parental cells, 110 genes showed down-regulation, with notable suppression observed in the MAPK pathway (*P* = 0.003) upon treatment with MRTX849 (Fig. 1F). The differential gene expression in MIA PaCa-2^R^ cells was reduced in response to MRTX849 compared to the parental cells (Fig. 1G). Before exposure to MRTX849, 1741 genes were found to be up-regulated (with a fold change of ≥2 and *q* < 0.05) in MIA PaCa-2^R^ cells (Fig. 1H). Subsequent gene set enrichment analysis (GSEA) using the Kyoto Encyclopedia of Genes and Genomes (KEGG) ranked the MAPK pathway as one of the top pathways in MIA PaCa-2^R^ cells compared to parental cells (Fig. 1I), in line with the involvement of the MAPK pathway in the response to G12Ci (*32*). The MAPK signaling pathway is a crucial signaling cascade that transmits signals from the cell surface to the nucleus in response to a variety of external stimuli, which plays a role in controlling fundamental cellular processes through a series of phosphorylation events (*33*).

The activation of MAPKs or the extracellular signal–regulated kinase (ERK) pathway is ignited by Ras guanosine triphosphatases to relay signals in different contexts, including during the regulation of cell proliferation and drug resistance (*34*). Immunoblots revealed that G12Ci-resistant cancer cells had higher phosphorylation of p-ERK1/2 at baseline. In the parental cells, MRTX849 completely inhibited ERK1/2 phosphorylation, but in the resistant cells, it only partially reduced ERK1/2 activation. In addition, the resistant cells demonstrated a more rapid reactivation of high levels of ERK activation when compared to the parental cells (Fig. 1J). These results indicate that the resistant *KRAS-G12C*–mutant cells have reactivated the MAPK signaling pathways.

### ABCC1 drives MRTX849-induced drug resistance

**Fig. 2.**
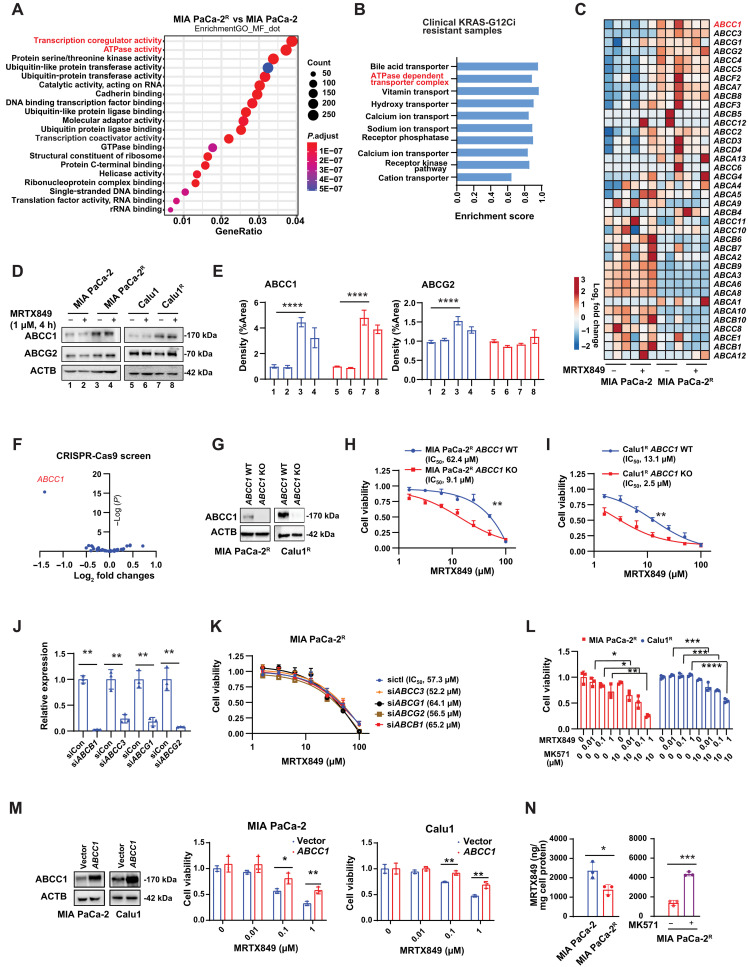
ABCC1 confers resistance in MRTX849-resistant cells. (**A**) GO enrichment of RNA-seq analysis. (**B**) GSEA was performed using a clinical tissue sample from an AMG510-resistant patient, available in a published database. A filtering step was applied to select gene sets associated with plasma membrane functions. (**C**) Heatmap showing the expression of ABC transporters in cells treated with MRTX849 (4 hours, 1 μM), based on RNA-seq. (**D** and **E**) Expression of ABCC1 and ABCG2 was examined by immunoblot analyses. Quantification was performed by the Image Lab software. (**F**) Partial volcano plot of 45 ABC transporters analyzed from genome-wide CRISPR-Cas9 screening. The *x* axis represents log_2_ fold changes of each gene. The *y* axis represents the negative logarithm of the *P* value. (**G** to **I**) MIA PaCa-2^R^ or Calu1^R^ and their *ABCC1* knockout (KO) cells were treated with MRTX849 followed by CCK-8 assays. (**J** and **K**) Small interfering RNA targeting *ABCB1*, *ABCC3*, *ABCG1*, and *ABCG2* was used for gene knockdown, followed by MRTX849 treatment and cell viability assays. The gene expressions in MIA PaCa-2^R^ cells were measured by quantitative PCR (Student’s *t* test). (**L**) Cells were treated with the ABCC1 inhibitor MK-571 and MRTX849 for 3 days, followed by CCK-8 assays. (**M**) Cells were transfected with an empty vector or ABCC1, treated with MRTX849 for 3 days, and subjected to CCK-8 assays. (**N**) Cells were treated with 1 μM MRTX849 for 6 hours or with 10 μM MK-571 for 24 hours, followed by 1 μM MRTX849 for 6 hours. Intracellular MRTX849 accumulation was quantified using LC-MS/MS (Student’s *t* test). The blots shown are representative of three independent experiments. Statistical analysis was performed using two-way ANOVA unless otherwise noted (*P < 0.05; **P < 0.01; ***P < 0.001; ****P < 0.0001). Data are presented as means ± SD, with *n* = 3 biologically independent samples.

Gene Ontology (GO) molecular function analysis of RNA sequencing (RNA-seq) enriches gene expression profiles by categorizing genes into functional groups, providing insights into the mechanisms underlying observed transcriptional changes. This analysis indicated that transcription coregulator activity and adenosine triphosphatase (ATPase) activity are among the top two differentially regulated molecular functions (Fig. 2A). ABC transporters are a large family of proteins that use ATP hydrolysis to transport a wide variety of substrates across cellular membranes, playing context-dependent roles in drug transport and resistance. ATPase activity is indispensable for ABC transporters that translocate substrates across biological membranes (*35*), hence favoring drug resistance (*36*).

An analysis was conducted on a patient diagnosed with *KRAS-G12C*–mutant lung adenocarcinoma who exhibited resistance to AMG510 therapy after 13 weeks of treatment (*37*). This patient’s tumor displayed resistance to both chemotherapy and immune checkpoint blockade as well (*37*). Through RNA-seq analysis of metastatic tumors in the neck lymph nodes during the AMG510-resistant phase compared to pretreatment lymph node tumors, a GO analysis revealed up-regulation of the ATPase-dependent transporter complex upon acquisition of resistance to AMG510 (Fig. 2B). Therefore, we focused on ABC transporters that are differentially expressed between MIA PaCa-2 and MIA PaCa-2^R^ cells. Several ABC transporters (e.g., *ABCC1*, *ABCC3*, *ABCG1*, and *ABCG2*) were up-regulated in MIA PaCa-2^R^ cells compared to parental cells (Fig. 2C). In MIA PaCa-2^R^ cells, elevated protein levels of ABCC1 and ABCG2 were observed compared to the parental cells. However, in Calu1^R^ cells, only ABCC1 exhibited increased expression, while the expression of ABCG2 remained unchanged compared to the parental cells (Fig. 2, D and E).

To identify the key ABC transporter responsible for MRTX849-induced resistance, we reanalyzed published data from a recent genome-wide CRISPR-Cas9 screen (*14*). This screen was conducted on parental H358 NSCLC cells (*KRAS-G12C* mutation) and their G12Ci-resistant derivatives (*KRAS-G12C*/*NRAS-Q61K* double mutation) (*14*). This screen identified *ABCC1* as a top-ranked gene whose disruption could reverse resistance to the G12Ci AMG510 (Fig. 2F). To determine whether ABCC1 is required for MRTX849-induced resistance, we knocked out *ABCC1* in MRTX849-resistant cells using CRISPR-Cas9 technology and established *ABCC1* knockout polyclonal (pooled) cell lines (Fig. 2G). The knockout of *ABCC1* sensitized MIA PaCa-2^R^ (Fig. 2H) and Calu1^R^ (Fig. 2I) cells to MRTX849. Of note, *ABCC1* knockout partially reversed resistance in MIA PaCa-2^R^ cells compared to parental cells, indicating that additional resistance mechanisms are involved in MIA PaCa-2^R^ cells. In contrast, the knockdown of *ABCB1*, *ABCC3*, *ABCG1*, or *ABCG2* failed to sensitize these cells to MRTX849 (Fig. 2, J and K). The ABCC1 inhibitor MK-571 (*38*) improved the efficacy of MRTX849 in two resistant cells (Fig. 2L). Furthermore, the overexpression of ABCC1 in two parental cells induced the resistance to MRTX849 (Fig. 2M). We also compared the intracellular accumulation of MRTX849 between parental and resistant cells. After 6 hours of MRTX849 treatment, resistant cells exhibited lower intracellular levels of MRTX849 compared to parental cells (Fig. 2N), suggesting active efflux of the drug from resistant cells. Treatment with the ABCC1 inhibitor MK-571 increased the intracellular content of MRTX849 (Fig. 2N). In conclusion, ABCC1 acts as a mediator of MRTX849-induced resistance.

### JUN mediates ABCC1 expression in MRTX849-induced resistance

**Fig. 3.**
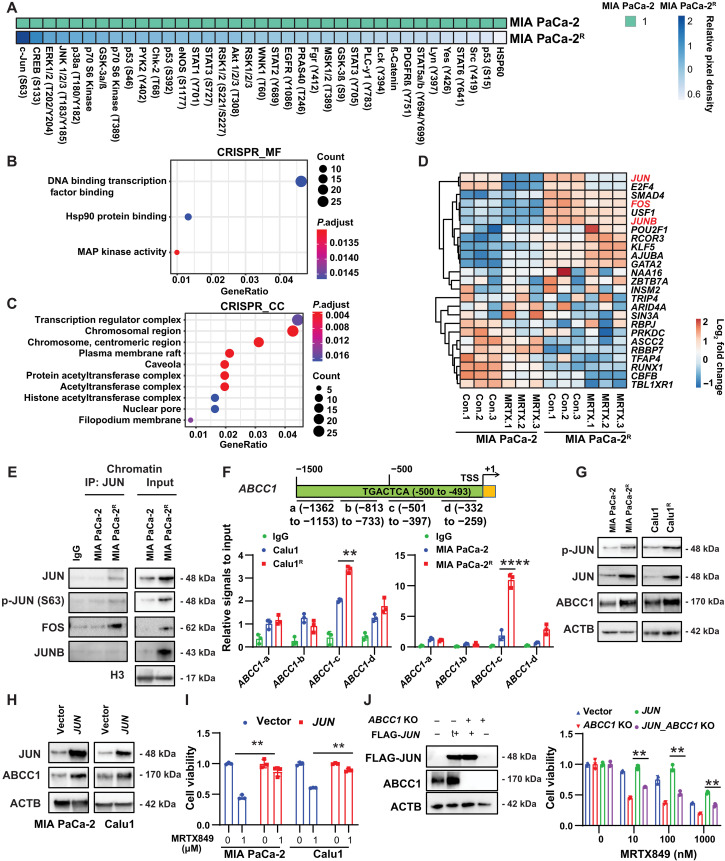
JUN positively regulates MRTX849-induced ABCC1 expression. (**A**) Heatmap of the phosphorylation of kinases in MIA PaCa-2 and MIA PaCa-2^R^ by human phosphokinase array. (**B** and **C**) GO terms of gene enrichment in the MF (B) and CC (C) categories of the 246 differentially KRAS-G12Ci resistance-associated genes from an analysis of a CRISPR screening. CC, cellular component; MF, molecular function. (**D**) Heatmap of the expression pattern of 25 transcription factors in MIA PaCa-2 and MIA PaCa-2^R^ in response to MRTX849 by next-generation sequencing. (**E**) Immunoprecipitation (IP) assay with anti-JUN antibodies using chromatin fraction in MIA PaCa-2 and MIA PaCa-2^R^ cells. (**F**) The ABCC promoter region is divided into a, b, c, and d fractions. ChIP assays were performed using digested chromatin from Calu1/Calu1^R^, MIA PaCa-2/MIA PaCa-2^R^, and anti-JUN antibodies. The binding DNA was purified. The ABCC1 promoter fractions a to d were measured by quantitative real-time PCR. TSS, Transcription Start Site. (**G**) Immunoblots of the indicated protein levels in MIA PaCa-2, MIA PaCa-2^R^, Calu1, and Calu1^R^ cells. (**H** and **I**) MIA PaCa-2 and Calu1 cells were transfected with pCDH-Flag-JUN and vector plasmid for 24 hours and then treated with MRTX849 for 3 days. The expression of the indicated proteins was shown by Western blot analysis. Cell viability was assessed. (**J**) Calu1 cells were infected with lentivirus expressing WT JUN followed by CRISPR-Cas9–mediated *ABCC1* knockout. The expression of ABCC1 and FLAG-JUN in Calu1 was measured by Western blot. Calu1 cells with JUN overexpression in the absence or presence of ABCC1 expression were exposed to the indicated drugs at the indicated concentrations for 72 hours, and cell viability was assessed. The blots shown are representative of three repeats. Statistical analysis was performed using two-way ANOVA (**P < 0.01; ****P < 0.0001). Data are presented as means ± SD, with *n* = 3 biologically independent samples.

Because protein phosphorylation is one of the most common and important posttranslational modifications involved in the control of KRAS signaling (*39*), we hypothesized that protein kinases might be involved in ABCC1 up-regulation. To explore this possibility, we measured the phosphorylation levels of 37 human protein kinases and their substrates in MIA PaCa-2^R^ cells by means of membrane-based antibody arrays, comparing them to their parental control cells. This procedure revealed G12Ci resistance-associated overphosphorylation of the activating protein 1 (AP1) transcription factor subunit JUN at Ser^63^ (Fig. 3A), which is critical for driving JUN-mediated gene transcription (*40*).

In parallel, we analyzed a genome-wide CRISPR-Cas9 screen (*14*), which found 246 genes as being depleted in G12Ci-resistant cells, but not in its parental cells, in response to G12Ci treatment. The GO analysis of the 246 genes identified from the CRISPR-Cas9 screen highlighted DNA transcription factor binding as a prominent category within the molecular function classification (Fig. 3B). In addition, transcriptional regulatory complexes, comprising 27 hits including *JUN*, *JUNB*, *FOS*, *CBFB*, *SMAD4*, *RUNX1*, *ZBTB7A*, *TFAP4*, *TBL1XR1*, *KLF5*, *RBPJ*, *AJUBA*, *AHR*, *ARID4A*, *E2F4*, *POU2F1*, *ASCC2*, *RCOR3*, *PRKDC*, *RBBP7*, *SIN3A*, *TRIP4*, *NAA16*, *INSM2*, *USF1*, *GATA2*, and *CREBBP*, were prominent in the cellular component category (Fig. 3C). Thus, G12Ci-resistant cells are characterized by abnormal transcription factor activity and gene expression.

Our RNA-seq analysis revealed differential expressions of *JUN*, *FOS*, and *JUNB* in MIA PaCa-2^R^ cells compared to MIA PaCa-2 cells (Fig. 3D). These encoded proteins belong to the AP1 complex, mainly composed of the JUN family (JUN, JUNB, and JUND) and FOS family (FOS, FOSB, FOSL1, and FOSL2) (*41*). Immunoprecipitation assays further demonstrated increased binding between JUN and FOS in the chromatin of MIA PaCa-2^R^ cells compared to MIA PaCa-2 cells (Fig. 3E), suggesting increased AP1 activation in MRTX849-resistant cells. In addition, we examined whether JUN is required for ABCC1 gene transcription. Chromatin immunoprecipitation (ChIP) assays showed increased binding of JUN protein to the *ABCC1* gene promoter region at positions −501 to −397 in the 5′ flanking region, in Calu1^R^ and MIA PaCa-2^R^ cells compared to Calu1 and MIA PaCa-2 cells (Fig. 3F). Consistently, an increased expression of p-JUN, JUN, and ABCC1 proteins were detected in MIA PaCa-2^R^ and Calu1^R^ cells compared to their parental cell lines (Fig. 3G). Functionally, transfection-enforced overexpression of JUN in parental cells stimulated the expression of ABCC1 and reduced growth inhibition in response to MRTX849 (Fig. 3, H and I). Conversely, the knockout of *ABCC1* abrogated the MRTX849-induced resistance by *JUN* overexpression (Fig. 3J), indicating that JUN-dependent ABCC1 expression drives MRTX849-induced resistance.

### Dasatinib enhances the efficacy of MRTX849 in MRTX849-resistant cells

**Fig. 4.**
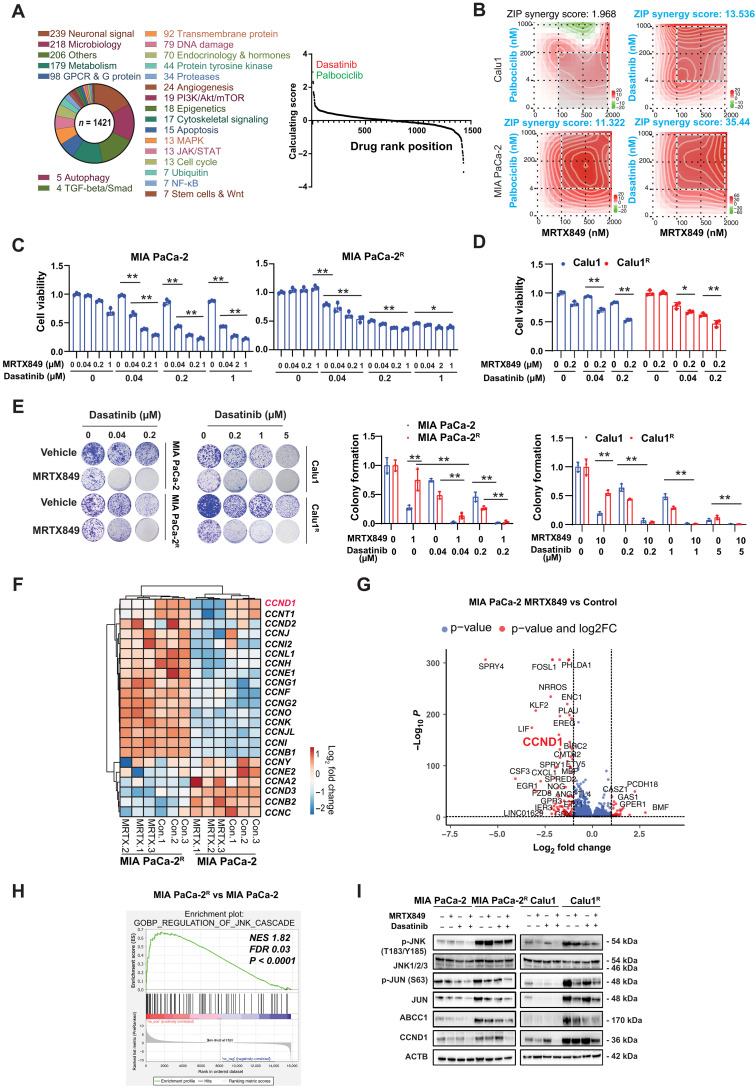
FDA-approved drug screen identifies that dasatinib inhibits JUN expression and enhances the efficacy of MRTX849. (**A**) Procedures of the US Food and Drug Administration (FDA)-approved drug screen. Pie chart of drugs with color codes corresponding to hit target type is shown. The calculating score is determined by taking log_2_ of the ratio of cell viability between drug-treated MIA PaCa-2 cells and drug-treated MIA PaCa-2^R^ cells. (**B**) ZIP synergy score was calculated by SynergyFinder (*43*) between dasatinib/palbociclib and MRTX849. (**C** and **D**) MIA PaCa-2/Calu1 and MIA PaCa-2^R^/Calu1^R^ cells were treated with MRTX849 and dasatinib for 3 days. Cell viability was measured by CCK-8 assay. (**E**) Cell clonogenic assay. MIA PaCa-2 and MIA PaCa-2^R^ cells were treated with the MRTX849 (1 μM) and dasatinib for 24 hours. Calu1 and Calu1^R^ cells were treated with the MRTX849 (10 μM) and dasatinib for 24 hours. All cells were observed for 10 days, followed by evaluation using crystal violet staining. Quantification was performed by ImageJ. (**F**) Heatmap showing the expression of the cyclin proteins in MIA PaCa-2 and MIA PaCa-2^R^ in response to MRTX849 by next-generation sequencing. (**G**) Volcano plot of differential gene expression between MRTX849 and vehicle in MIA PaCa-2 (absolute fold change of ≥2; *q* value <0.05). *CCND1* was substantially down-regulated. (**H**) GSEA showing that the JNK signaling pathway is up-regulated between MIA PaCa-2^R^ and MIA PaCa-2 cells (nominal *P* value of <0.0001). (**I**) Immunoblot analysis of the indicated proteins of cells treated with 1 μM dasatinib and 1 μM MRTX849 for 24 hours. The blots shown are representative of three independent experiments. Statistical analysis was performed using two-way ANOVA unless otherwise noted (*P < 0.05; **P < 0.01; ***P < 0.001). Data are presented as means ± SD, with *n* = 3 biologically independent samples.

We next sought to identify drugs that overcome the acquired resistance to MRTX849. We performed a combinatorial drug screening to evaluate the synergistic interaction of MRTX849 with a panel of 1421 FDA-approved drugs in MIA PaCa-2 and MIA PaCa-2^R^ cells (Fig. 4A). We found that 63 drugs targeting 19 kinases [including 9 protein tyrosine kinases, 4 kinases from the Janus kinase–signal transducers and activators of transcription (JAK-STAT) pathway, 3 MAPK kinases, and 3 kinases involved in the phosphatidylinositol 3-kinase (PI3K)–Akt–mammalian target of rapamycin (mTOR) pathway] restored MRTX849-mediated growth inhibition. Among them, dasatinib and palbociclib had a greater growth inhibitory effect on MIA PaCa-2^R^ than MIA PaCa-2 cells (Fig. 4A). In addition, we conducted a gene set analysis using DSigDB (*42*), a drug signatures database, on the differentially expressed genes identified in RNA-seq data from the resistant cells compared to the parental cells. This method enabled the exploration of candidate drugs for potential therapeutic interventions against MRTX849-resistant cells. Through this approach, dasatinib and bosutinib emerged as notable candidates within the databases (fig. S1B).

To validate these results, we compared the synergy score of dasatinib/MRTX849 and palbociclib/MRTX849 on growth inhibition through SynergyFinder, a stand-alone web application for interactive analysis and visualization of drug combination screening data (*43*). The combination of dasatinib and MRTX849 displayed a higher synergistic score compared to that of palbociclib and MRTX849 in MIA PaCa-2 and Calu1 cells (Fig. 4B). The combination of G12Ci and palbociclib is currently being evaluated in preclinical studies (*11*) and clinical trials (ClinicalTrials.gov identifier: NCT04185883). We decided to prioritize the dasatinib/MRTX849 combination, which displayed synergistic interactions in the suppression of tumor cell viability (Fig. 4, C and D) and colony formation (Fig. 4E). While Calu1^R^ cells exhibited resistance compared to parental Calu1 cells, MIA PaCa-2^R^ cells display an even greater level of resistance compared with parental MIA PaCa-2 cells. This disparity in resistance may stem from the intrinsic differences between these two cell lines.

G12Ci resistance is associated with cell cycle reactivation driven by cyclins, a family of proteins necessary for cell cycle synthesis. We analyzed the expression of 22 cyclin family genes in MIA PaCa-2 and MIA PaCa-2^R^ cells in response to MRTX849 from next-generation sequencing data (Fig. 4F). Cyclin D1 (*CCND1*) was among the top cyclin family genes that were up-regulated in MIA PaCa-2^R^ cells (Fig. 4F). *CCND1* was down-regulated in response to MRTX849 in parental MIA PaCa cells (Fig. 4G). GSEA analysis indicated that the c-Jun N-terminal kinase (JNK) signaling pathway was up-regulated in MIA PaCa-2^R^ compared to MIA PaCa-2 cells (Fig. 4H), which was consistent with the immunoblots showing JNK activation in both types of resistant cells (Calu1^R^ and MIA PaCa-2^R^) (Fig. 4I). In addition, immunoblot analysis showed that the protein expression of CCND1 (but not of CCNA2, CCNB1, or CCNE1) was augmented in MIA PaCa-2^R^ and Calu1^R^ cells (Fig. 4I and fig. S1C). ChIP assays revealed increased binding of JUN protein to the *CCND1* gene promoter region (at positions −1073 to −893) in Calu1^R^ and MIA PaCa-2^R^ cells compared to their counterparts (fig. S1D), indicating that JUN is involved in the differential transactivation of *CCND1*. The effect of MRTX849 on the cell cycle is time dependent (fig. S1, E and F). After 4 hours of treatment, no significant change was observed in the cell cycle between parental and resistant cells. However, a notable difference emerged at 24 hours. Furthermore, both cell lines displayed a G_1_ phase cell cycle arrest within 4 to 24 hours following treatment with a combination of MRTX849 and dasatinib (fig. S1, E and F). The dasatinib/MRTX849 combination inhibited CCND1 expression and decreased ABCC1 expression, which was associated with the inactivation of JNK and JUN at the protein (Fig. 4I) and mRNA (fig. S1, G to I) levels.

The combination of dasatinib and MRTX849 resulted in greater growth inhibition than either treatment alone across 11 *KRAS-G12C*–mutant NSCLC cell lines (SW873, H358, HCC44, HCC4019, DFCI024, HCC2122, H1373, H1792, H2030, HOP62, and UM-UC-3). Conversely, no significant difference was observed in the *KRAS-G12D*–mutant cell line PANC1 and *KRAS* WT cell lines (fig. S2A). Moreover, the combination of MRTX849 and dasatinib reduced colony formation in all 11 KRAS-G12C–mutant cell lines (fig. S2B). These findings collectively demonstrate that the combination of dasatinib and MRTX849 effectively overcomes G12Ci-induced resistance.

### SRC promotes MRTX849-induced ABCC1 expression

**Fig. 5.**
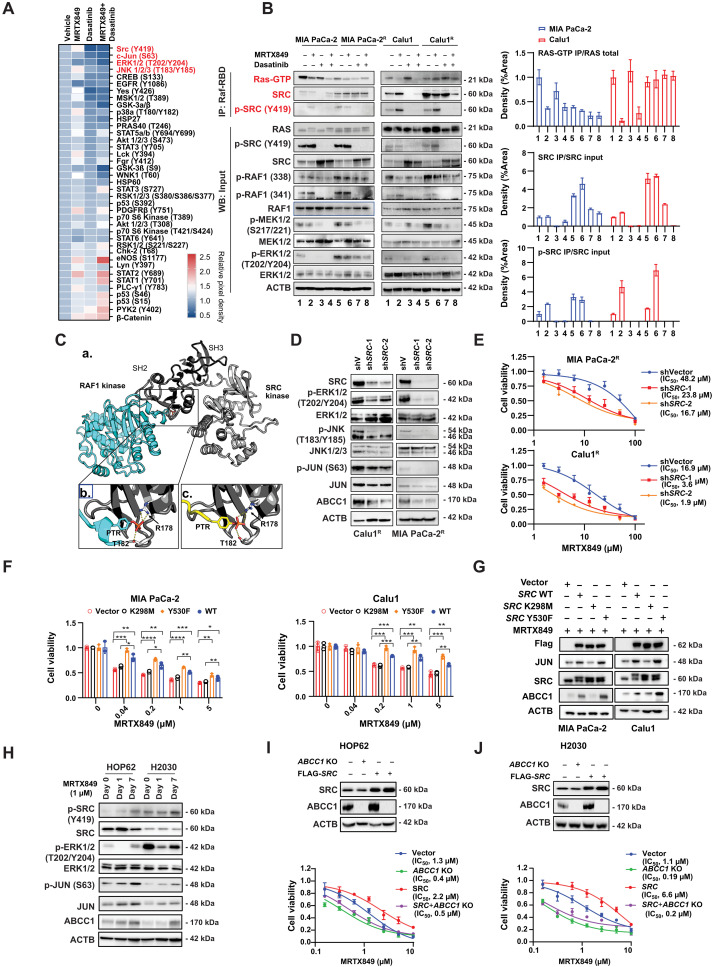
SRC positively regulates MRTX849-induced multidrug resistance. (**A**) MIA PaCa-2 and MIA PaCa-2^R^ cells were treated with dasatinib (1 μM, 24 hours) and MRTX849 (1 μM, 48 hours), followed by human phosphokinase array and quantification. (**B**) Cells were treated with 1 μM dasatinib and 1 μM MRTX849 for 24 hours, followed by RAS-GTP pull-down assays, Western blotting, and quantification. (**C**) Model of phosphorylated RAF1 bound to the SH2. (a) Structure of the computer-generated model: RAF1 (based on AlphaFold AF-P04049-F1) and SRC (based on the PDB). (b) SH2 (PDB: 1FMK) complexed with phosphorylated tyrosine pentapeptide Y341 (RAF1), interacting with Arg^178^ and Thr^182^ of SRC. (c) Human SH2 (PDB: 1HCS, dark gray) complexed with Y341 (yellow). (**D**) Immunoblot analysis of gene knockdown in Calu1^R^ and MIA PaCa-2^R^ cells. (**E**) Cells were treated with MRTX849 for 3 days and then assessed by CCK-8 assays. (**F**) Cells were transfected with an empty vector, SRC-WT, SRC-K298M (kinase dead), or SRC-Y530F (active form), treated with drugs for 3 days, and assessed by CCK-8 assays. (**G**) Cells were transfected with the indicated plasmids, treated with MRTX849 (1 μM) for 3 days and analyzed by immunoblotting. (**H**) HOP62 and H2030 cells were treated with MRTX849 (1 μM) for 1 and 7 days, followed by immunoblots. (**I** and **J**) *ABCC1* WT and knockout cells of HOP62 and H2030 cells were transfected with an empty vector or SRC-WT-Flag and treated with MRTX849 for 3 days, followed by CCK-8 assays. The blots shown are representative of three independent experiments. Statistical analysis was performed using two-way ANOVA unless otherwise noted (*P < 0.05; **P < 0.01; ***P < 0.001; ****P < 0.0001). Data are presented as means ± SD, with *n* = 3 biologically independent samples.

Dasatinib is a multitarget kinase inhibitor that is used in the therapy of chronic myelogenous leukemia (*44*, *45*) and acute lymphoblastic leukemia (*46*). Phosphokinase arrays confirmed that dasatinib inhibited the phosphorylation of multiple kinases, including SRC, JUN, ERK1/2, JNK, CREB, EGFR, YES, and MSK1/2 in MIA PaCa-2^R^ cells (Fig. 5A). G12Ci can lock K-RAS-G12C in its inactive GDP-bound conformation, thereby inhibiting KRAS-dependent RAF-MEK-ERK signaling (*47*). RAS-GTP pull-down assays revealed that the binding of RAS-GTP to the RAS binding domain (RBD) of RAF1 was sufficiently repressed by MRTX849, but not by dasatinib, in parental cells (Fig. 5B). Although RAS-GTP binding to RAF1-RBD was hindered, RAF1 bound more SRC in MIA PaCa-2^R^ and Calu1^R^ cells, suggesting that SRC may substitute for RAS to activate RAF and MAPK signaling in MRTX849-resistant cells. These findings are consistent with the observed elevation in RAF phosphorylation at S338 and Y341 in the resistant cells, along with the activation of downstream signaling pathways such as MEK and ERK (Fig. 5B). Molecular docking analysis proposed a model indicating potential interactions between phosphorylated tyrosine (PTR341) of RAF1 and Arg^178^ and Thr^182^ residues of SRC [Fig. 5C (see a and b)]. Notably, RAF1 Y341 resides within a YEEI consensus motif for Src homology 2 domain (SH2) binding. The YEEI motif (Tyr-Glu-Glu-Ile) in SRC is part of the regulatory C-terminal tail (Y530 in human SRC) (*48*). Similar interactions have also been seen in numerous experimental structures of the SH2 bound to phosphorylated peptides; this is exemplified by an nuclear magnetic resonance (NMR) structure shown for comparison [Protein Data Bank (PDB) ID: 1HCS; Fig. 5B (see c)]. Immunoprecipitation experiments also revealed a higher binding efficiency of RAF1 to SRC in the resistant cells compared to the parental cells (fig. S3A).

Because FDA-approved SRC inhibitors (such as dasatinib and bosutinib) are multitargeted kinase inhibitors, we assessed DGY-06-116, a selective irreversible covalent inhibitor of SRC (*49*), designed to target a unique cysteine residue in the P-loop of SRC (*49*, *50*). This specificity allows DGY-06-116 to covalently bind to SRC, resulting in high selectivity and potency. DGY-06-116 targets kinases such as SRC, YES1, and ABL1, with less pronounced effects on HCK, LCK, LYN, and BTK. Unlike dasatinib, DGY-06-116 shows minimal activity against c-KIT, EphA2, and PDGFR (*49*). We found that dasatinib and DGY-06-116 significantly enhanced the effect of MRTX849, while JNK inhibitors (such as JNK inhibitor IX and JNK-In-8) also increase MRTX849’s growth inhibition of MIA PaCa-2^R^ cells, albeit to a lesser degree (fig. S3B). This supports the partial involvement of the SRC-JNK pathway in MRTX849 resistance. We also assessed the effects of DGY-06-116, dasatinib, and an MEK inhibitor (trametinib) on signal transduction pathways in both MIA PaCa-2^R^ and Calu1^R^ cells. DGY-06-116 and dasatinib exhibited suppression of SRC activation, p-RAF1 (Y341)/p-MEK(S217/221)/p-ERK(T202/Y204), and p-JUN(S63)/JUN, as well as down-regulation of ABCC1 expression (fig. S3C). In contrast, trametinib specifically inhibited the activation of ERK1/2 but did not affect the phosphorylation or expression of JUN and ABCC1 (fig. S3C). These findings suggest that targeting ERK alone may not be sufficient to suppress the JUN/ABCC1 pathway.

Subsequently, we assessed DGY-06-116 for its potential to synergize with MRTX849. The combination of MRTX849 and DGY-06-116 demonstrated synergistic efficacy against *KRAS-G12C* cell lines (DFCI024, HCC44, HCC4017, H1373, H358, Calu1, and SW873), while no synergy was observed against the *KRAS* WT cell line HCC2279 (fig. S3D). In addition, the combination of MRTX849 and DGY-06-116 induced a synergistic effect in colony formation across *KRAS-G12C*–mutant cell lines but not in KRAS WT cells (fig. S3, E and F). Furthermore, knockdown of *SRC* using two specific short hairpin RNAs confirmed the critical role of SRC in promoting the activation of ERK and JUN, as well as the expression of ABCC1 in Calu1^R^ and MIA PaCa-2^R^ cells (Fig. 5D). The knockdown of *SRC* in Calu1^R^ and MIA PaCa-2^R^ also confers sensitivity to MRTX849 (Fig. 5E).

Next, we investigated the impact of ectopic expression of SRC WT, SRC K298M [a kinase-dead form of SRC (*51*)], and SRC Y530F [the activated form of SRC (*51*)] in MIA PaCa-2 and Calu1 cell lines treated with MRTX849. Our results demonstrated that SRC WT and Y530F, but not K298M, drive sensitive cells to become resistant to MRTX849 (Fig. 5F), as evidenced by elevated expression of JUN and ABCC1 (Fig. 5G).

We investigated the impact of MRTX849 treatments (1 μM for 1 and 7 days) on the SRC/JUN/ABCC1 pathways in *KRAS-G12C*–mutant NSCLC cell lines HOP62 and H2030. Our findings revealed that a 1-day treatment inhibited ERK activation; however, a 7-day treatment led to the reactivation of ERK associated with an increase in the SRC/JUN/ABCC1 pathway (Fig. 5H). Overexpression of *SRC* induced resistance to MRTX849, whereas knockout of *ABCC1* in these two cell lines increased sensitivity to MRTX849. Furthermore, *ABCC1* knockout abolished the resistance conferred by *SRC* overexpression in response to MRTX849, highlighting the pivotal role of ABCC1 in mediating SRC-mediated resistance to MRTX849 (Fig. 5, I and J).

### The anticancer effect of MRTX849 with SRC inhibitors in KRAS-G12C cell-line derived mouse models

**Fig. 6.**
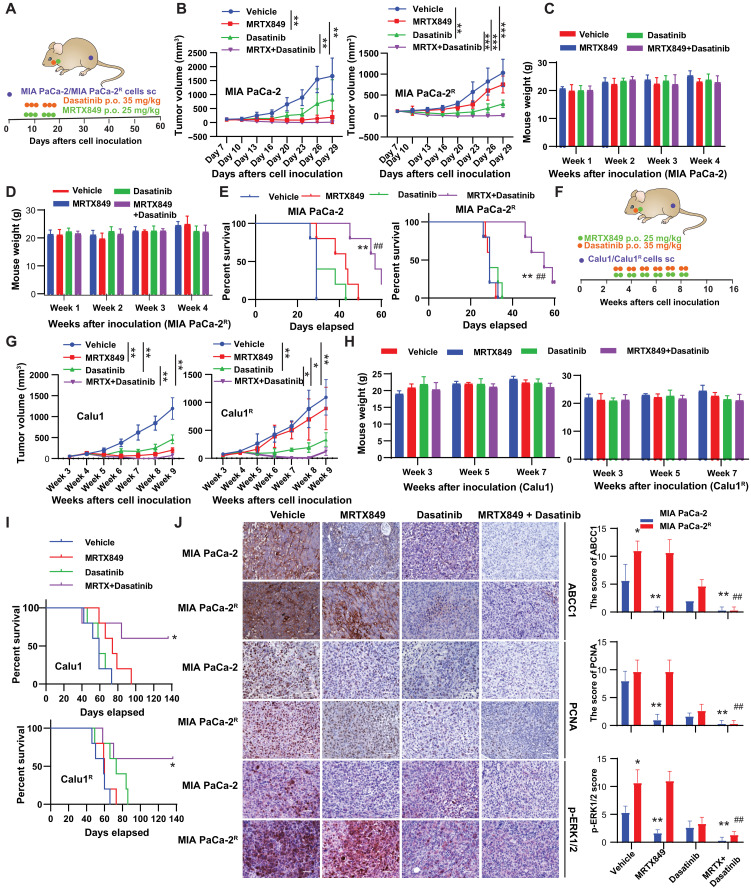
Synergistic effect of MRTX849 and dasatinib on tumor suppression in mouse models. (**A**) Schematic diagram of the therapeutic strategy of the combination of MRTX849 (35 mg/kg, p.o., oral gavage) and dasatinib (35 mg/kg, oral gavage) in MIA PaCa-2/MIA PaCa-2^R^ xenograft models. sc, subcutaneously. (**B**) The tumor growth was monitored twice per week (*n* = 8 to 10 tumor; **P < 0.01; ***P < 0.001, two-way ANOVA). (**C** and **D**) Body weight was monitored after the administration of MRTX849 and dasatinib in nude mice for the indicated weeks after MIA PaCa-2/MIA PaCa-2^R^ inoculation. (**E**) Survival curve of each group [*n* = 5 mice per group; **P < 0.01 compared with the vehicle; ##*P* < 0.01 compared with MRTX849 treatment, log-rank (Mantel-Cox) test]. (**F**) Schematic diagram of the therapeutic strategy of the combination of MRTX849 (25 mg/kg, p.o., twice a week for 6 weeks) and dasatinib (35 mg/kg, p.o., twice a week for 6 weeks) in Calu1/Calu1^R^ xenograft models. (**G**) Tumor growth was monitored twice per week (*n* = 8 to 10 tumors). (**H**) Body weight was monitored after the administration of MRTX849 and dasatinib in nude mice for the indicated weeks after Calu1/Calu1^R^ inoculation. (**I**) Survival curve of each group [*n* = 5 mice per group; *P < 0.05, log-rank (Mantel-Cox) test]. (**J**) Staining of ABCC1, p-ERK1/2, and PCNA in MIA PaCa-2/MIA PaCa-2^R^ xenograft models on day 29. Scale bars, 50 μm. Quantification of immunohistochemistry analysis was performed by calculation using the immunoreactive score (*79*) (*n* = 3; *P < 0.05 or **P < 0.01 compared with the vehicle; ##*P* < 0.01 compared with MRTX849 treatment).

We examined the efficacy and safety of dasatinib and MRTX849 in immunodeficient mice engrafted with parental MIA PaCa-2 and MRTX849-resistant MIA PaCa-2^R^ cells. The dosages for MRTX849 and dasatinib were determined through a literature review (*11*, *52*, *53*). This methodology aimed to strike an optimal balance between maximizing therapeutic efficacy and minimizing toxicity. Tumors were allowed to progress to 80 to 100 mm^3^ of volume and then treated with six cycles of oral gavage with MRTX849 or dasatinib, alone or in combination in the xenografts of MIA PaCa-2 and MIA PaCa-2^R^ (Fig. 6A). MRTX849 alone caused rapid tumor shrinkage in MIA PaCa-2 cell xenografts but failed to suppress the growth of MRTX849-resistant xenograft tumors. In contrast, MRTX849 combined with dasatinib caused the MRTX849-resistant xenograft tumors to shrink (Fig. 6B and fig. S4A). Increased toxicity, including body weight loss, was not observed following the combination treatment compared to either of the monotherapies (Fig. 6, C and D, and fig. S4B). The combination substantially prolonged the survival of mice bearing xenografts of MIA PaCa-2 and MIA PaCa-2^R^ (Fig. 6E). Similar results were obtained in mice receiving Calu1 or Calu1^R^ xenografts that were treated with 12 cycles of MRTX849 and/or dasatinib (Fig. 6, F to I, and fig. S4C). While dasatinib reduces tumor growth, the lack of significant change in survival can be attributed to several factors. Tumor growth inhibition does not always correlate with improved overall survival due to the tumor microenvironment and host responses. In addition, the timing and duration of dasatinib treatment may affect survival outcomes. Immunohistochemistry confirmed that MRTX849 plus dasatinib reduced the expression of p-ERK1/2, ABCC1, and the cell proliferation marker proliferating cell nuclear antigen (PCNA) in the tumors (Fig. 6J and fig. S4D). Furthermore, phosphorylation of RAF1 at Y341 (p-RAF1) was increased in Calu1^R^ and MIA PaCa-2^R^ xenografts but markedly inhibited upon treatment with dasatinib alone (fig. S4E).

**Fig. 7.**
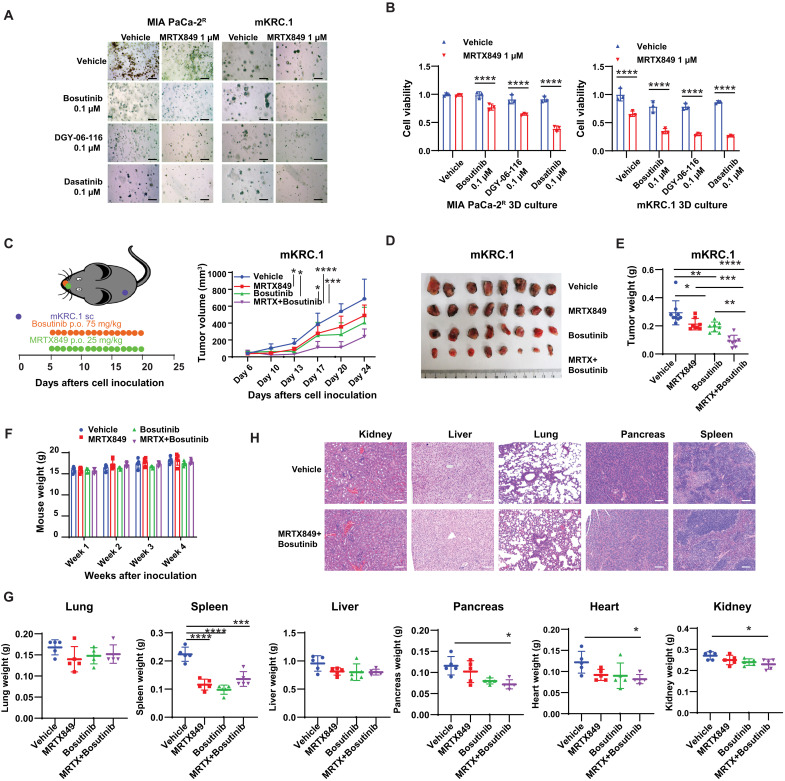
Efficacy and safety profile of the combination of MRTX849 and bosutinib in a KRAS G12C murine model mKRC.1. (**A**) Bright-field microscopy images of 3D sphere cultures of MIA PaCa-2^R^ and mKRC.1 with vehicles, MRTX849, dasatinib, DGY-06-116, and bosutinib as well as combinations, after 7 days of treatment. Scale bars, 500 μm. (**B**) Measurement of cell viability in 3D sphere cultures of MIA PaCa-2^R^ and mKRC.1 with different treatments (*n* = 3 biologically independent samples; ****P < 0.0001, two-way ANOVA). (**C**) Schematic diagram of the therapeutic strategy of the combination of MRTX849 (35 mg/kg, oral gavage, p.o.) and bosutinib (75 mg/kg, oral gavage) in mKRC.1 syngeneic mouse models. The tumor growth was monitored twice per week (*n* = 8 to 10 tumors; *P < 0.05; ***P < 0.001; ****P < 0.0001, two-way ANOVA with Tukey’s multiple comparisons test). (**D**) Photo of tumors on day 27. (**E**) Tumor weight on day 27. *n* = 8 tumor per group. *P < 0.05; **P < 0.01; ***P < 0.001; ****P < 0.0001, two-way ANOVA. (**F**) Body weight was monitored after the treatments for the indicated weeks. *n* = 5 mice per group; two-way ANOVA. (**G**) Organ weight in each group was measured on day 27 (*n* = 5; *P < 0.05; ***P < 0.001; ****P < 0.0001, two-way ANOVA). (**H**) Representative images of H&E staining of the liver, lung, pancreas, and spleen from mice bearing mKRC.1 tumors, treated with either vehicle or the combination of MRTX849 and bosutinib. Scale bars, 50 μm.

We further evaluated the impact of combination therapy on three-dimensional (3D) sphere cultures of MIA PaCa-2^R^ and mKRC.1 [a murine KRAS-G12C line derived from a tumor in a genetically engineered mouse model (*54*)]. In MIA PaCa-2^R^ cells, dasatinib demonstrated a superior synergistic effect compared to either bosutinib or DGY-06-116 (Fig. 7, A and B). Furthermore, the combined use of bosutinib, DGY-06-116, or dasatinib with MRTX849 demonstrated a similar synergistic effect in mKRC.1 cells (Fig. 7, A and B).

SRC inhibitors have been associated with various adverse events, including bleeding, infection, anemia, thrombocytopenia, diarrhea, edema, pleural effusion, and pulmonary hypertension (*55*). Subsequently, we investigated the efficacy and safety of bosutinib and MRTX849 in C57BL/6 mice engrafted with mKRC.1 cells. Tumors were allowed to progress to a volume of 80 to 100 mm^3^ before undergoing 16 cycles of oral gavage with MRTX849 or bosutinib, either alone or in combination. As stand-alone treatments, MRTX849 or bosutinib resulted in minimal growth inhibition. However, their combination notably inhibited tumor growth (Fig. 7, C to E). No notable body weight loss was observed following combination treatment compared to either monotherapy (Fig. 7F). However, there was a decrease in spleen, pancreas, heart, and kidney weight in the combination group (Fig. 7G). Furthermore, the combination group exhibited enlarged glomeruli, disorganized renal tubules in the kidneys, and inflammatory infiltration in the pancreas (Fig. 7H). In the control group, spleen weight increased, likely due to tumor progression. This increase was mitigated in the combination treatment group (Fig. 7G). In addition, the treated group showed an expansion of white pulp, a major control center for the humoral immune response (*56*), in the spleens compared to the control group, as evidenced by hematoxylin and eosin (H&E) staining (Fig. 7H). Although the combination therapy of bosutinib and MRTX849 did not lead to significant body weight loss, implying a certain degree of tolerability, it may have potential effects on the spleen, pancreas, heart, and kidneys. Therefore, careful dosing management and adverse reaction control are crucial in balancing efficacy and safety in these combination therapies.

### ABCC1 up-regulation in MRTX849-resistant cells leads to multidrug resistance

**Fig. 8.**
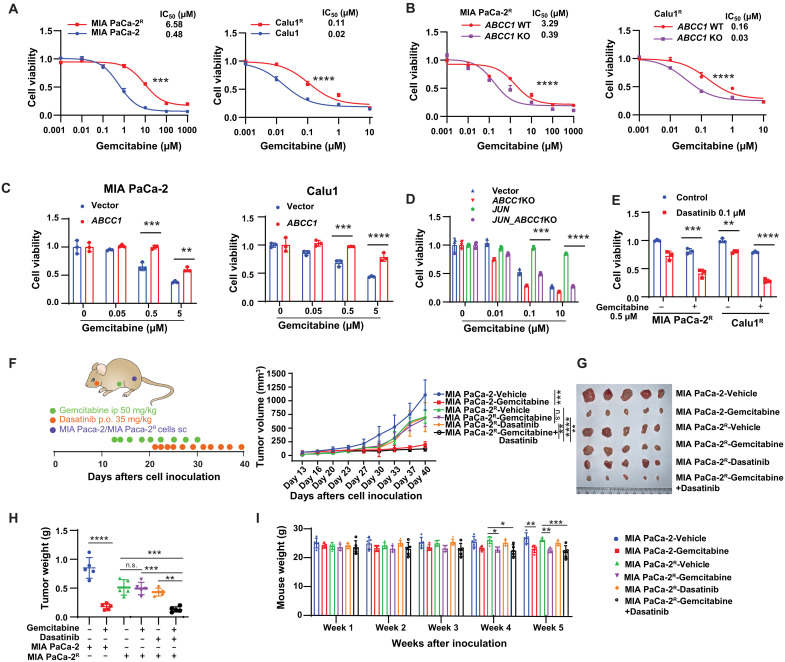
ABCC1 up-regulation in MRTX849-resistant cells leads to gemcitabine resistance. (**A**) Calu1/Calu1^R^ and MIA PaCa-2/MIA PaCa-2^R^ cells were treated with gemcitabine for 4 days at the indicated concentrations. The percentage of viable cells is shown relative to untreated controls by CCK-8 assays. (**B**) Calu1^R^ and MIA PaCa-2^R^ *ABCC1* WT and knockout cells were treated with gemcitabine for 2 days at the indicated concentrations. The percentage of viable cells is shown relative to untreated controls by CCK-8 assays. (**C**) Calu1 and MIA PaCa-2 cells were transfected with *ABCC1* or vector and then treated with gemcitabine for 2 days at the indicated concentrations. The percentage of viable cells is shown relative to untreated controls by CCK-8 assays. (**D**) Calu1 cells were infected with lentivirus expressing WT JUN followed by CRISPR-Cas9–mediated *ABCC1* knockout. The cells were treated with gemcitabine for 2 days followed by CCK-8 cell viability assays. (**E**) Calu1^R^ and MIA PaCa-2^R^ cells were treated with gemcitabine in the presence or absence of dasatinib for 3 days followed by cell viability assays. (**F**) Schematic diagram of the therapeutic strategy of gemcitabine and dasatinib in MIA PaCa-2/MIA PaCa-2^R^ xenograft models. The tumor growth was monitored twice per week (*n* = 5 mice). ip, intraperitoneally. (**G**) Photo of the tumors on day 42. (**H**) Tumor weight on day 42 (*n* = 5). (**I**) Body weight was monitored in nude mice for the indicated weeks after MIA PaCa-2/MIA PaCa-2^R^ inoculation (*n* = 5). Statistical analysis was performed using two-way ANOVA (*P < 0.05; **P < 0.01; ***P < 0.001; ****P < 0.0001). *n* = 3 biologically independent samples unless otherwise specified. n.s., not significant.

We also investigated whether MRTX849-induced ABCC1 up-regulation affects the sensitivity of gemcitabine, a commonly used chemotherapy drug in the treatment of advanced NSCLC and pancreatic cancer. We observed that gemcitabine resistance is increased in MIA PaCa-2^R^ and Calu1^R^ cells compared to their counterparts (Fig. 8A). *ABCC1* knockout in the resistant cells conferred sensitivity to gemcitabine (Fig. 8B). In addition, *ABCC1* overexpression in MIA PaCa-2 and Calu1 cells led to resistance against gemcitabine (Fig. 8C). Transfection-induced overexpression of *JUN* in Calu1 cells induced resistance to gemcitabine (Fig. 8D). Conversely, the knockout of *ABCC1* reversed the resistance to gemcitabine induced by *JUN* overexpression (Fig. 8D), indicating that JUN-dependent ABCC1 expression contributes to gemcitabine-induced resistance. Furthermore, dasatinib enhanced the efficacy of gemcitabine in MIA PaCa-2^R^ and Calu1^R^ cells (Fig. 8E).

Subsequently, we assessed the efficacy of gemcitabine in immunocompromised murine models engrafted with either parental MIA PaCa-2 or MRTX849-resistant MIA PaCa-2^R^ cells. Tumors were allowed to progress to a volume of 80 to 100 mm^3^ before being treated with five cycles of gemcitabine. We observed that MIA PaCa-2^R^ xenografts exhibited resistance to gemcitabine compared to MIA PaCa-2 xenografts. Consequently, we treated these mice with 10 cycles of dasatinib, which diminished both tumor growth and weight (Fig. 8, F to H). We noted a decrease in mouse weight beginning in week 4 in both the gemcitabine and the combination treatment groups. However, there was no significant difference in the reduction in tumor weight between the combination treatment and gemcitabine alone (Fig. 8I), suggesting that the toxicity was due to gemcitabine. These findings suggest that the up-regulation of ABCC1 in MRTX849-resistant cells leads to subsequent multidrug resistance.

A recent paper demonstrated nongenetic mechanisms of resistance to AMG510 involving integrin β4 and found that AMG510-resistant lung cancer cell lines (H358, H23, and SW1573) remain sensitive to MRTX849 (*57*). We found that MRTX849-resistant cell lines (MIA PaCa-2^R^ and Calu1^R^) exhibit resistance to AMG510 (fig. S5, A and B). This underscores that the mechanisms of resistance to these two G12Ci can differ depending on the context.

### The synergistic anticancer effect of MRTX849 with SRC inhibitors in KRAS-G12C patient-derived organoids

**Fig. 9.**
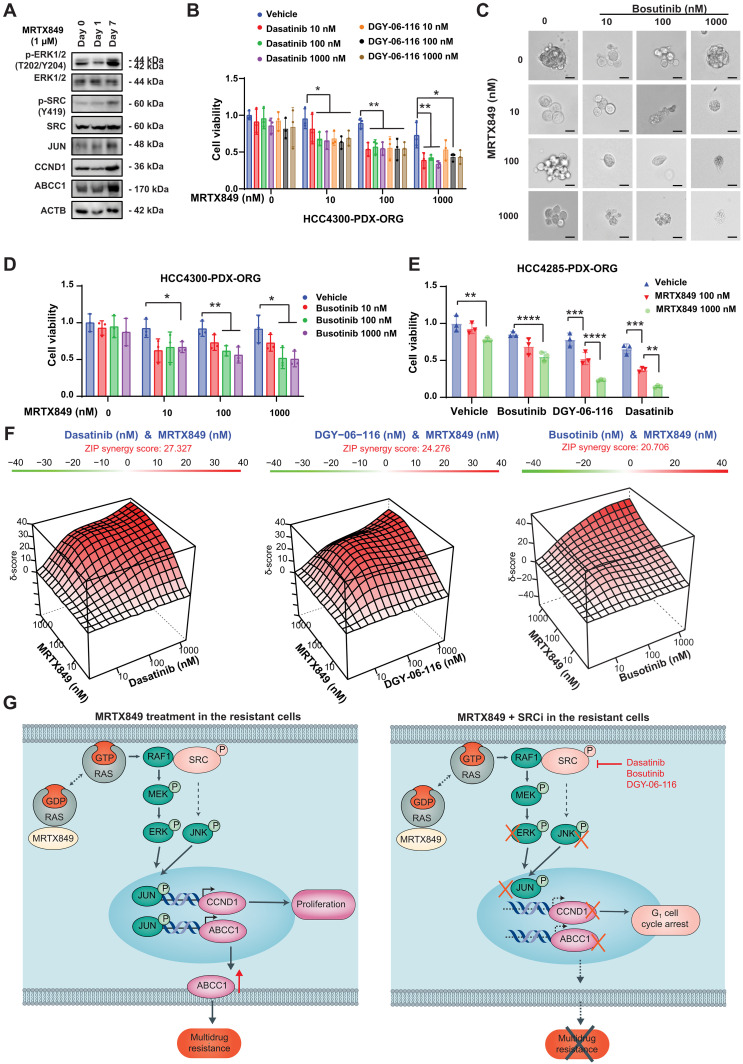
Effects of MRTX849 and SRC inhibitors in the treatment of *KRAS-G12C*–mutant patient-derived organoids. (**A**) Patient-derived organoids HCC4300-PDX-ORG were treated with MRTX849 for 1 or 7 days, and the expression of the indicated proteins was measured. The blots shown are representative of three repeats. (**B**) Patient-derived organoids HCC4300-PDX-ORG treated with vehicle, MRTX849, dasatinib, or DGY-06-116, as well as combinations, after 7 days of treatment. Cell viability of the organoids was measured by CCK-8. (**C**) Bright-field microscopy images of HCC4300-PDX-ORG treated with vehicle, MRTX849, or bosutinib, as well as combinations, after 7 days of treatment. Scale bars, 50 μm. (**D**) HCC4300-PDX-ORG was treated with vehicle, MRTX849, or bosutinib, as well as combinations, after 7 days of treatment. Cell viability of the organoids was measured by CCK-8. (**E**) HCC4285-PDX-ORG was treated with vehicle, MRTX849, bosutinib, DGY-06-116, or dasatinib, as well as combinations, after 7 days of treatment. Cell viability of the organoids was measured by CCK-8. (**F**) 3D synergy map displaying ZIP synergy score between MRTX849 and dasatinib, DGY-06-116, or bosutinib in HCC4300-PDX-ORG. (**G**) Proposed mechanisms and strategies to overcome MRTX849-induced multidrug resistance. In G12Ci-resistant cells, SRC binding with RAF1 bypasses the inhibition of KRAS-G12C by MRTX849, resulting in JUN activation and subsequent ABCC1 and CCND1 expression, and confers a multidrug-resistant phenotype and uncontrolled cell proliferation. Dasatinib, bosutinib, or DGY-06-116 suppresses the phosphorylation of SRC on Y416 and thus inhibits JUN activation and the expression of ABCC1 and CCND1, preventing or reversing G12Ci-induced multidrug resistance and inducing G_1_ cell cycle arrest. Statistical analysis was performed using two-way ANOVA (*P < 0.05; **P < 0.01; ***P < 0.001; ****P < 0.0001). *n* = 3 biologically independent samples unless otherwise specified.

To determine the clinical relevance of our findings, we investigated the response to MRTX849 for a shorter time (1 day) and longer time (7 days) in an established patient-derived organoid model (HCC4300-PDX-ORG) with *KRAS-G12C* mutation. The ERK activation was reduced at day 1 but increased in day 7, which was associated with increasing SRC phosphorylation, JUN expression, as well as the expression of CCND1 and ABCC1 (Fig. 9A), indicating the activation of SRC/JUN/ABCC1 and SRC/JUN/CCND1 pathways. Our findings indicated that single-agent MRTX849 or a SRC inhibitor, including dasatinib, DGY-06-116, and bosutinib, did not significantly affect the growth of the organoids. However, the combination of MRTX849 and an SRC inhibitor demonstrated a robust suppressive effect on the growth of HCC4300-PDX-ORG (Fig. 9, B to D, and fig. S6A). In another KRAS-G12C mutant organoid, HCC4285-PDX-ORG, single treatments with MRTX849, dasatinib, DGY-06-116, and bosutinib had a modest effect on growth inhibition. However, combination treatments significantly reduced organoid growth (Fig. 9E and fig. S6B). The combination of MRTX849 and a SRC inhibitor, including dasatinib, DGY-06-116, and bosutinib yielded high synergy scores in HCC4300-PDX-ORG (Fig. 9F). These studies reveal that long-time treatment with MRTX849 increases the ABCC1 expression and diminishes the efficacy of MRTX849 in KRAS-G12C patient-derived organoids. The addition of SRC inhibitors can enhance the anticancer effect of MRTX849.

## DISCUSSION

Given the tremendous and long-awaited success for the development of G12Ci therapies, it is important to decipher and overcome resistance mechanisms that may limit their potential. In this study, G12Ci-resistant cancer cells displayed a more rapid reactivation of high levels of ERK activation compared to parental cells. However, they still exhibited responsiveness to G12Ci at an early time point, suggesting that G12Ci could still effectively bind KRAS, and the resistance mechanism was unlikely to be attributed to a mutation in the KRAS binding pocket (Fig. 1J). Mechanistically, we identified and established a substantial role for the tyrosine protein kinase SRC in driving G12Ci-induced multidrug resistance by stimulating JUN-mediated transactivation of genes encoding the multidrug resistance–associated protein ABCC1 and the cell cycle driver CCND1 (Fig. 9G). The inhibition of SRC and JUN enhanced the antitumor activity of MRTX849 and reversed the multidrug-resistant phenotype. These insights may lead to the development of previously unidentified combination treatment strategies aimed at enhancing the therapeutic efficacy of G12Ci in patients.

A common mechanism of multidrug resistance consists in the overexpression of cancer drug–extruding ABC transporters in malignant cells (*58*). To date, at least 15 human ABC superfamily transporters, including ABCB1 (*59*), ABCC1 (*60*), and ABCG2 (*61*), have been reported to mediate multidrug resistance in different circumstances, including G12Ci treatment (*27*, *62*, *63*). A recent study highlighted the suppressive effects of adagrasib on ABCB1 but not on ABCG2 functionality, using short-term treatment (up to 3 days) to assess ABCB1 activity and demonstrating adagrasib’s effectiveness (*64*). In contrast, our research extends over a longer period (7 days to 6 months), revealing that resistant cells exhibit a distinct phenotype compared to those observed after shorter treatment durations. Therefore, various members of the ABC family may have a context-dependent role in mediating multidrug resistance, influenced by not only tumor and drug type but also treatment duration.

In the specific context of MRTX849-induced multidrug resistance, the relevant efflux pump is ABCC1, as demonstrated here by using transcriptomic and bioinformatic analyses of CRISPR-Cas9 screens. ABCC1, which was originally cloned from the human small cell lung cancer cell line H69AR (*60*), is known to transport a variety of endogenous substrates, such as the antioxidant glutathione and pro-inflammatory leukotrienes (*65*). The overexpression of ABCC1 in tumor cells has been implicated in the multidrug-resistant phenotype caused by cytotoxic drugs (*60*). As polymorphisms affecting ABCC1 have also been associated with drug responses (*66*), the characterization of genetic variations affecting ABCC1 in patients with G12Ci might yield previously unidentified predictive biomarkers.

Our study identifies the SRC-JUN pathway as another target for overcoming G12Ci resistance. In this pathway, SRC can mediate JUN activation through the RAF1-MEK-ERK pathway, but JNK activation may also contribute to JUN phosphorylation. Defining the cross-talk between RAS and MAPK signaling (ERK and JNK) is key to understanding the upstream signaling mechanisms of tolerance to G12Ci therapy. Regardless, we show here that the transcription factor AP1 member JUN is critical for the transactivation of at least two genes (*ABCC1* and *CCND1*) involved in G12Ci resistance. It is conceivable that mutations in the KRAS vertical signaling pathway could lead to SRC binding to RAF1, thereby altering SRC signaling and activating JUN-mediated gene transcription regulation. Furthermore, receptor tyrosine kinases such as EGFR, FGFR, and other kinases like protein kinase C, may activate SRC signaling and produce similar outcomes (*67*). In addition, targeting KRAS-G12C may lead to hyperactivation of focal adhesion kinase (FAK), thereby triggering FAK/SRC signaling and activating the JNK/ERK pathway (*68*). These studies offer valuable insights into the potential pathways underlying the activation of the SRC-JUN-ABCC1 pathway upon treatment with G12Ci. Further research is needed to investigate the relationship between SRC activation and other reported G12Ci resistance mechanisms.

Now, direct pharmacological inhibition of JUN remains a challenge. However, pharmacological inhibition of upstream activators of JUN, especially JNK kinases, is a feasible strategy. Likewise, small-molecule inhibition of ABCC1 has so far failed in clinical trials, necessitating alternative methods for preventing or attenuating ABCC1 up-regulation (*69*). The nonreceptor tyrosine kinase SRC, the product of the first described proto-oncogene, plays a critical role in mediating various signal transduction pathways (*70*). The KRAS/MAPK and SRC pathways are interconnected (*71*), but their relative contribution to G12Ci responses and G12Ci resistance remained elusive. Here, we demonstrate that multiple reversible pharmacological SRC inhibitors, as well as the knockdown of SRC, can restore the anticancer activity of MRTX849 in drug-resistant cells. Among a thousand clinically used drugs, dasatinib turned out to be the most effective in enhancing therapeutic MRTX849 responses as well as in subverting MRTX849-induced multidrug resistance. Dasatinib and bosutinib have anticancer activity against NSCLC (*72*, *73*). Mechanistically, the combination of MRTX849 and dasatinib inhibited JUN-dependent expression of ABCC1 and CCND1 in multidrug-resistant cancer cells. The combination therapy of dasatinib or bosutinib with MRTX849 effectively eliminated MRTX849-resistant *KRAS-G12C*–mutant cancer cells in mouse models or organoid models, with minimal observed side effects. Careful dosing management is pivotal in achieving the optimal balance between efficacy and safety in these combination therapies.

We also demonstrated that an irreversible covalent inhibitor of SRC, DGY-06-116, which targets cysteine-277 in the P-loop (*49*, *50*), enhances the antitumor activity of MRTX849 in multiple KRAS-G12C cancers. Although it remains to be proved, for *KRAS-G12C*–mutated tumors that express high levels of SRC, optimal therapeutic responses may be obtained by the combination of MRTX849 (or other G12Ci inhibitors), DGY-06-116 (or other SRC inhibitors), and perhaps additional cancer drugs affecting mechanistically distinct circuitries (such as immunotherapies).

At present, there are ~30 active clinical trials involving G12Ci MRTX849 or AMG510 in *KRAS-G12C*–mutant cancers. While some trials, such as NCT03785249 (*28*), NCT03600883 (*9*), and NCT04303780 (*74*), have been conducted, the occurrence of acquired resistance to KRAS-G12Ci has been confirmed in some cases. Various putative mechanisms of resistance have been identified, including both on-target and off-target mechanisms that can confer resistance to KRAS-G12Ci (*13*). In addition, some patients selected for these trials may have preexisting multidrug resistance (*9*, *37*, *74*), which could contribute to treatment failure. Our findings suggest that up-regulation of ABCC1 contributes to the acquired resistance observed in our resistant model, although it is likely not the sole underlying mechanism. While the knockout of *ABCC1* augmented the sensitivity of the cells to G12Ci, it did not have the same effect as observed in the sensitive cells. Further exploration is necessary to determine additional mechanisms that may contribute to acquired resistance to KRAS-G12Ci.

In summary, we have identified and characterized the mechanisms of multidrug resistance elicited by G12Ci. Our preclinical results also suggest that targeting the multidrug resistance–relevant SRC-JUN-ABCC1 pathway involved in multidrug resistance may create previously unidentified opportunities to improve the therapeutic response of *KRAS-G12C*–mutated tumors treated with G12Ci.

## MATERIALS AND METHODS

### Cell lines and cell culture

The human cancer cell lines Calu1 (a human NSCLC cell line), MIA PaCa-2 (a human PDAC cell line), UM-UC-3 (a human urinary bladder carcinoma cell line), PANC1 (a human PDAC cell line), HT1080 (a human fibrosarcoma cell line), and SW873 (a human colorectal carcinoma cell line) were purchased from the American Type Culture Collection. 293FT was purchased from Thermo Fisher Scientific (R70007). These cell lines were cultured in Dulbecco’s modified Eagle’s medium (DMEM) with 10% fetal bovine serum (FBS). Other NSCLC cell lines, including H358, HCC44, HCC4019, DFCI024, HCC2122, H1373, H1792, H2030, HOP62, and HCC2279, were obtained from the Hamon Center cell repository of UT Southwestern Medical Center. In addition, mKRC.1 [a murine KRAS-G12C line derived from a tumor in a genetically engineered mouse model (*54*)] was obtained from L.E.H. These cells were cultured in RPMI 1640 supplemented with 5% FBS.

MIA PaCa-2 cells carry a *KRAS* mutation (G12C), a *TP53* mutation, a homozygous deletion of *p16 (CDKN2A)*, and are WT for *SMAD4* (*75*). According to the Catalogue Of Somatic Mutations In Cancer (COSMIC), Calu1 cells have the following mutations (*76*): *HOXA13* (*p.Q288R*), *KRAS* (*p.G12C*), *APC* (*p.R1171C*), *DNMT3A* (*p.R38C*), *TRIP11* (*p.M422V*), *SETBP1* (*p.N1288K*), *TERT* (*p.V791I*), *PTPRB* (*p.M2164V*), *EZR* (*p.K253R*), *ROS1* (*p.R317W*), *SPEN* (*p.S2532N*), *NIN* (*p.S1837T*), *LRP1B* (*p.S2851I*), *NFE2L2* (*p.P128L*), *ABL2* (*p.K959R*), and *HNF1A* (*p.A161T*). Although Calu1 cells also harbor a KEAP1 mutation (*p.P128L*) (*77*), this mutation is not listed in COSMIC. Cell line identity was confirmed by DNA fingerprinting (Promega, PowerPlex 1.2 Kit) and mycoplasma-free status was verified by polymerase chain reaction (PCR) (Boca Scientific, e-Myco Kit). The above cell lines were grown in an incubator maintained at 37°C and 5% CO_2_.

Organoid cells derived from HCC4300-PDX-ORG (KRAS-G12C) and HCC4285-PDX-ORG (KRAS-G12C) were established at UT Southwestern Medical Center. Both of them were obtained from treatment-naïve patients diagnosed with lung adenocarcinoma. The cells were seeded in growth factor–reduced Matrigel (Corning) domes and cultured in a complete human feeding medium containing advanced DMEM/F12 (Invitrogen, 12634-010), 1× B27 supplement (Invitrogen, 17504-044), N2 (Invitrogen, 17502-048), 10 mM Hepes, 0.01 μM GlutaMAX, 10 mM nicotinamide (Sigma-Aldrich, N0636), 1 mM *N*-acetylcysteine (Sigma-Aldrich, A9165), human EGF (5 ng/ml; PeproTech, AF-100-15), human FGF-10 (20 ng/ml; PeproTech, 100-26), R-Spondin 3 (250 ng/ml; R&D Systems, 3500-RS), 5 nM Neuregulin 1/Heregulin β-1 (PeproTech, 100-03), FGF-7 (5 ng/ml; PeproTech, 100-19), noggin (100 ng/ml; PeproTech, 120-10C), 500 nM SB 202190 (Sigma-Aldrich, S7067), 500 nM A83-01 (Tocris, 2939), and 5 μM Y27632 (Abmole, M1817). The organoids were regularly fed with this medium to support their growth and maintenance.

### Reagents

MRTX849 (S8884), JNK inhibitor IX (S7508), JNK-IN-8 (S4901), bosutinib (S1014), PP1 (S7060), and PP2 (S7008) were purchased from Selleckchem. Dasatinib (HY-10181), bosutinib (HY-10158), DGY-06-116 (HY-136605), MK2206 (HY-10358), gemcitabine (HY-17026), MRTX1133 (HY-134813), and MK-571 sodium (HY-19989A) were purchased from MedChemExpress. DSS (disuccinimidyl suberate) was obtained from Thermo Fisher Scientific (21655). A human phosphokinase array kit was purchased from R&D Systems (ARY003C).

Antibodies directed against the following were used at a concentration of 1:1000 unless noted otherwise. The following antibodies were from Cell Signaling Technology: p-MEK1/2 (S217/221; 9154), MEK1/2 (9122; 4694), ERK1/2 (9102), p-ERK (4370), RAS (8832), KRAS (33197), SRC (2109), p-RAF1 (Ser338; 9427), PCNA (2586), p-JNK (9252), JNK (9251), RAF1 (12552), p-SRC (Tyr416; 2101), ACTB (3700), MRP1/ABCC1 (14685), p-JUN (S63; 91952), JUN (9165), FOS (4384), JUNB (3753), H3 (4499), and CCND1 (2978). The antibody to p-RAF1 (Y341) was purchased from Abcam (ab59223). ABCB1 (A19093) and ABCG2 (A5661) were purchased from Abclonal.

CRISPR ABCC1 guide RNA lentiviral transduction particles were purchased from Sigma-Aldrich (clone ID: HSPD0000026420; plasmid sequence: ATCTCTCCCGACAGACCG). Our pMIEG3–c-Jun was obtained from Addgene (40348). Our pCDH-Flag-JUN was created by the Tang lab. ABCC1 cDNA ORF (open reading frame) clone was purchased from Sino Biological (HG17448-UT). pDEST40-2XFL-SRC (140295), pDEST40-2XFL-Src-K298M (140315), and pDEST40-2XFL-Src-Y530F (140319) were purchased from Addgene.

### Cell viability assay

Cells were seeded into 96-well plates and incubated with the indicated treatments. Subsequently, 100 μl of a fresh medium was added to cells containing 10 μl of Cell Counting Kit-8 (CCK-8) solutions (Biomake, B34304) and incubated for 2 hours (37°C, 5% CO_2_). Absorbance at 450 nm was measured using a microplate reader (BioTek, Cytation 5 Cell Imaging Multi-Mode Reader).

### Colony formation assay

Related cells were plated into a 6-well plate or 24-well plate at 100 to 2000 cells per well and treated as indicated for 6 to 10 days. Then, the media were aspirated, and cells were fixed with cold methanol for 10 min. Fixed cells were stained with crystal violet (0.05%) for 30 min at room temperature. The dye was washed off with water, and the plates were image scanned after drying.

### Cellular MRTX849 accumulation assay

To initiate the experiment, cells were seeded in triplicate in 6-well plates with ~500,000 cells per well. After treatment, cells were washed, harvested using trypsin, and counted. Each sample was diluted in cold PBS to achieve a final concentration of 1 million cells/ml and then frozen using liquid nitrogen. The frozen samples were stored at −80°C until liquid chromatography–tandem mass spectrometry (LC-MS/MS) analysis (*78*). To create a standard curve, blank lysates were spiked with varying concentrations of each compound. Next, 200 μl of methanol containing 0.15% (final concentration of 0.1%) formic acid, 3 mM NH_4_ acetate (2 mM final concentration), and 150 ng/ml (final concentration of 100 ng/ml) *N*-benzylbenzamide was added to each sample, which was vortexed for 15 s, incubated at room temperature for 10 min, and spun twice at 13,200 rpm in a standard microcentrifuge. The supernatant was then subjected to LC-MS/MS analysis. The cell pellets were also preserved for protein concentration determination. Specifically, they were resuspended in 100 μl of 0.1 N NaOH and heated to 95°C for 5 min, and 5 μl of the resulting solution was mixed with 5 μl of a bovine serum albumin (BSA) standard curve in a 96-well plate. Then, 200 μl of the Pierce Bicinchoninic acid (BCA) protein reagent was added to the plate, which was incubated for 30 min at 37°C before measuring the absorbance at 562 nM. Last, the concentrations of the samples were interpolated using the BSA standard curve, and the concentrations of the compounds in the lysates were normalized to the protein content of each sample.

### Western blot

Cells were lysed with a 1× cell lysis buffer (Cell Signaling Technology, 9803) containing a protease inhibitor on ice for 10 min, homogenized by passing through a 21-gauge needle, and centrifuged at 14,000*g* for 15 min at 4°C to pellet the cell debris. Proteins were quantified using a BCA assay (Thermo Fisher Scientific, 23225), and 20 μg of each sample was resolved on 4 to 12% Criterion XT Bis-Tris gels (Bio-Rad) in an XT MES running buffer (Bio-Rad, 1610789) and transferred to polyvinylidene difluoride membranes (pore size: 0.22 μM; Bio-Rad, 1620233) using the Trans-Blot Turbo Transfer Pack and System (Bio-Rad). Membranes were blocked with Tris-buffered saline with Tween 20 (TBST) containing 5% skim milk for 1 hour and incubated overnight at 4°C with various primary antibodies. Following three washes in TBST, membranes were incubated with goat anti-rabbit/mouse immunoglobulin G (IgG) horseradish peroxidase (HRP) secondary antibodies (1:3000; Cell Signaling Technology, 7074 or 7076) at room temperature for 1 hour and washed. Chemiluminescence substrate was applied using the SuperSignal West Pico Chemiluminescent Substrate (Thermo Fisher Scientific, 34080) or SuperSignal West Femto Maximum Sensitivity Substrate (Thermo Fisher Scientific, 34095), and blots were analyzed using the ChemiDoc Touch Imaging System (Bio-Rad). Image Lab Software (Bio-Rad, version 6.1.0) was used for view and analysis.

### Quantitative real-time PCR

Total RNA was extracted and purified from cultured cells using the RNeasy Plus Mini Kit (QIAGEN, 74136) according to the manufacturer’s instructions. The RNA was quantified by determining the absorbance at 260 nm. One microgram of total RNA from each sample was reverse transcribed into cDNA using the iScript cDNA synthesis kit (Bio-Rad, 170-8891) in a volume of 20 μl. The cDNA from cell samples was amplified. Quantitative real-time PCR was performed using the ssoFast EvaGreen Supermix (Bio-Rad, 172-5204) on the C1000 Touch Thermocycler CFX96 Real-Time System (Bio-Rad) according to the manufacturer’s protocol. Analysis was performed using the Bio-Rad CFX Manager software (Bio-Rad). The primers, which were synthesized and desalted from Sigma-Aldrich, are as follows: *ABCC1*-F, CCGTGTACTCCAACGCTGACAT; *ABCC1*-R, ATGCTGTGCGTGACCAAGATCC; *CCND1*-F, TCTACACCGACAACTCCATCCG; *CCND1*-R, TCTGGCATTTTGGAGAGGAAGTG; *JUN*-F, CCTTGAAAGCTCAGAACTCGGAG; *JUN*-R, GCTGCGTTAGCATGAGTTGGC; *ABCG1*-F, GAGGGATTTGGGTCTGAACTGC; *ABCG1*-R, TCTCACCAGCCGACTGTTCTGA>; *ABCC3*-F, GAGGAGAAAGCAGCCATTGGCA; *ABCC3*-R, TCCAATGGCAGCCGCACTTTGA; *ABCG2*-F, GTTCTCAGCAGCTCTTCGGCTT; *ABCG2*-R, TCCTCCAGACACACCACGGATA; *ABCB1*-F, GCTGTCAAGGAAGCCAATGCCT; *ABCB1*-R, TGCAATGGCGATCCTCTGCTTC; *GAPDH*-F, GTCTCCTCTGACTTCAACAGCG; and *GAPDH*-R, ACCACCCTGTTGCTG-TAGCCAA.

### Coimmunoprecipitation

To extract protein lysates from cultured cells, a lysis buffer (Cell Signaling Technology, 9803) supplemented with a protease inhibitor mixture and phosphatase inhibitor mixture (Roche, 11873580001 and 04906845001) was used. Chromatin factions were isolated by a chromatin extraction kit (Abcam, ab117152). Specific antibodies were mixed with Dynabeads (Invitrogen, 14311D) and then incubated for at least 16 hours on a rotator. Protein concentrations were adjusted to 2 μg/μl. The mixture of proteins and Dynabeads containing the required antibodies was incubated on a rotator at 4°C for 1 hour. After being washed three times with Phosphate-Buffered Saline with Tween 20 (PBS-T), the mixtures were resuspended with an SDS loading buffer. After a boiling water bath for 10 min and centrifugation at 4°C for 3 min, the Dynabeads were obsoleted, and the supernatant was used for further analysis.

### Ras-GTP pull-down assay

Ras-GTP was pulled down using the Ras Activation Assay Biochem Kit (Cytoskeleton, BK008) according to manufacturer’s instructions. Briefly, cells were lysed with a cell lysis buffer and total protein was quantified using a BCA Protein Assay Kit (Pierce, 23225). Following quantification, 2 mg of protein was incubated with Raf-RBD beads, rotating at 4°C for 2 hours. Bead protein complexes were collected and washed before being resuspended in a 1× Laemmli buffer and boiled for 2 min at 95°C. The samples were then analyzed by SDS–polyacrylamide gel electrophoresis and Western blot analysis with a RAS-specific antibody (Cell Signaling Technology, 8821).

### FDA-approved drugs screening

A panel of 1421 drugs (Selleckchem, L1300) was applied to MIA PaCa-2 and MIA PaCa-2^R^ cells at 10 μM in the presence or absence of MRTX849 at a fixed dose of 1 μM. Cell viability was determined using CCK-8 after 3 days of drug treatment. For primary hit discovery, we identified synergistic combinations based on the value of the cell viability of MRTX849 versus that of the combination. To refine the list of hits, we used a scoring system based on the cell viability of the drug in MIA PaCa-2 versus MIA PaCa-2^R^ cells. The top combination was validated using SynergyFinder 2.0.

### Cell cycle analysis

Cells were fixed with 70% ethanol overnight, washed, and resuspended in 300 to 500 μl of a propidium iodide (PI)/Triton X-100 staining solution. A 10-ml staining solution was made of 0.1% (v/v) Triton X-100 (Sigma-Aldrich, X100) and 2 mg of DNase-free RNase A (Sigma-Aldrich, 11119915001) and 0.40 ml of PI (500 μg/ml; Sigma-Aldrich, P4170). Cells were first gated using forward scatter (FSC)/side scatter (SCC) to exclude the debris. FSC-H/FSC-A and SSC-H/SSC-A gates were used to identify single cells and eliminate doublets from the analysis. Data were acquired on a BD Accuri C6 Flow Cytometer (BD Biosciences). Data were analyzed by the BD Accuri C6 Plus software (version 1.0.27.1). G_0_-G_1_ and G_2_-M phase histogram peaks were separated by an S-phase distribution. The FSC and SCC were used to identify single cells. Analysis was performed using a 532-nm excitation with a 585/40-nm bandpass filter.

### ChIP assay

ChIP assays were performed in accordance with a SimpleChIP Plus Enzymatic Chromatin IP Kit (Cell Signaling Technology, 9005). Briefly, Calu1 and Calu1^R^ cells, grown to near 80% confluence, were fixed with formaldehyde and lysed. Chromatin was fragmented by partial digestion with micrococcal nuclease to obtain chromatin fragments of one to five nucleosomes. ChIPs were performed using c-Jun Rabbit mAb (Cell Signaling Technology, 9165) or Normal Rabbit IgG (Cell Signaling Technology, 2729) and ChIP-Grade Protein G Magnetic Beads. After a reversal of protein-DNA cross-links, the DNA was purified using DNA purification spin columns, which were analyzed by quantitative real-time PCR. The JUN binding site was amplified by PCR using the input DNA (1%) or DNA isolated from precipitated chromatin as templates and primers flanking the putative *ABCC1* or *CCND1* binding sites in their promoters. The primers were as follows: *ABCC1*-a-F (−1362 to −1153), ATAGACTTCCCAC-CTCCCTG; *ABCC1*-a-R (−1362 to −1153), GCATTCACAGTGACAAGGCT; *ABCC1*-b-F (−813 to −733), TGTCTCCAGGCTTCAGTTTCC; *ABCC1*-b-R (−813 to −733), AGAGCGC-CTTTGCTTCTTCT; *ABCC1*-c-F (−498 to −397), ACTCAGCTTTGGAGTCAGCG; *ABCC1*-c-R (−498 to −397), CCAGGTGCAGAGAGGTTGAG; *ABCC1*-d-F (−332 to −259), TACAC-TCCAGGCAGGTAGGG; *ABCC1*-d-R (−332 to −259), ACCCT-GCGACCACTTTTCAA; *CCND1*-a-F (−1497 to −1398), CATCTTGCTGTGAGCACCCT; *CCND1*-a-R (−1497 to −1398), TCAGTGTCATCAAACCACCGT; *CCND1*-b-F (−1073 to −893), GACGTCTACACCCCCAACAA; *CCND1*-b-R (−1073 to −893), CGGGAGAAACACACCTCTGA; *CCND1*-c-F (−844 to −645), CAAGGACCGACTGGTCAAGG; *CCND1*-c-R (−844 to −645), GTCGTTGCAAATGCCCAAGG; *CCND1*-d-F (−664 to −465), CCTTGGGCATTTGCAACGAC; and *CCND1*-d-R (−664 to −465), GCATTTCCAAGAACGCCACG.

### Next-generation sequencing

An Agilent 2100 Bioanalyzer system and RNA Nano chip kit (5067-1511) were used for RNA quality measurement. An Illumina TruSeq Stranded mRNA Library prep kit (20020594) was used to generate the RNA libraries. The first step in the workflow involved purifying the poly-A containing mRNA molecules using oligo-dT attached magnetic beads. Following purification, the mRNA was fragmented into small pieces using divalent cations under elevated temperature. The cleaved RNA fragments were copied into first-strand cDNA using reverse transcriptase and random primers. Second-strand cDNA synthesis followed, using DNA polymerase I and RNase H. The cDNA fragments then went through an end repair process, the addition of a single “A” base, and then ligation of the adapters. The products were then purified and enriched with PCR to create the final cDNA library. AMPure XP beads (Beckman Coulter, A63881) purifications were performed two times before the assessment took place. The library quantity was measured using a PicoGreen method. A Quant-iT PicoGreen dsDNA Assay kit (Invitrogen, P7589) and PerkinElmer plate reader (PerkinElmer Victor X3, 2030 Multilabel Reader) were used in the assessment. The library quality was verified on an Agilent 2100 Bioanalyzer instrument using an Agilent DNA 1000 kit (5067-1504). All libraries must have met the quality control (QC) requirements before we moved forward with sequencing.

The samples were sequenced on an Illumina NovaSeq 6000 sequencer platform with S4 flowcell and XP workflow PE-150. Adapter trimming and quality trimming were performed with Trim Galore (v0.6.4), and ribosomal RNA (rRNA) was removed using SortMeRNA (v2.1b). Trimmed and filtered reads were aligned to reference (GRCh37) with STAR (vSTAR2.6.1d). FeatureCounts (v1.6.4) was used for gene counts, biotype counts, and rRNA estimation Fragments Per Kilobase of transcript per Million mapped reads (FPKMs) for genes and transcripts were generated by StringTie (v2.0), and RSeQC (v3.0.1) was used for generating RNA QC metrics. The differential gene analysis, GO, and KEGG pathway enrichment analyses were performed by RStudio (2021.09.0) and iDEP-96. GSEA was performed to analyze the data.

### Proteome profiler antibody array analysis

The R&D Systems Human Phospho-Kinase Array Kit (ARY003C) consists of membrane-based sandwich immunoassays that are used. Captured antibodies spotted in duplicate on nitrocellulose membranes bond to specific target proteins present in the sample (step 1). Captured proteins were detected with biotinylated detection antibodies (step 2) and then visualized using chemiluminescent detection reagents (step 3). The signal produced was proportional to the amount of analyte bound. The intensities were analyzed with the Quick Spots Image Analysis Software (Western Vision Software; http://wvision.com/QuickSpots.html).

### Structure modeling and docking analysis

To investigate the structural basis of the binding of SRC and RAF1, the crystal structure of the human tyrosine-protein kinase (SRC; PDB ID: 1FMK) at 1.5-Å resolution was used for docking studies. The structure includes SH2, SH3, and kinase domain. For RAF1, an AlphaFold model (AF-P04049-F1) was chosen because it showed high-confidence modeling of residues at the N terminus next to Y341, which are truncated in other structures available at the PDB. Before protein preparation and docking, RAF1 Y341 was phosphorylated to phosphotyrosine (PTR) using the mutate residue function in Schrödinger’s Maestro. All PDB-obtained structures were loaded and prepared using the Protein Preparation Wizard tool integrated in Schrödinger’s Maestro 2022. All protein structures were preprocessed using Prime by adding missing hydrogens, correcting the bond order assignment, adding missing side chains, and adjusting charges. As part of this, protonation states of amino acids such as His, Glu, and Asp were optimized for pH 7.2. Last, restrained minimization was performed using an OPLS4 force field as a final energy minimization for hydrogen bond optimization. PIPER was used for the protein-protein docking experiments to generate an SRC-RAF1 complex model. PIPER uses a global search by means of a fast Fourier transform approach, which can evaluate a substantial number of poses in an efficient way with reduced false-positive results. Typically, comparable poses are generated and scored according to the simple atomistic energy function that can effectively identify potential relative poses. First, by default, PIPER evaluated 70,000 poses throughout the protein-protein docking and kept the top 1000 poses based on their scores for the next step. Second, the 1000 selected poses were clustered according to their common structural features. Last, the best poses from the clusters were obtained and ranked based on the cluster size. From the results, we prioritized models that showed similar interactions between the SH2 and the PTR, as compared to interactions with the ~70 experimental structures in the PDB. The solution NMR structure of the SH2 complexed with a phosphorylated tyrosine pentapeptide (PDB ID: 1HCS) was representative of a typical interaction.

### Preclinical models with acquired resistance to G12Ci treatment

We conducted all animal care and experiments in accordance with guidelines from the Association for Assessment and Accreditation of Laboratory Animal Care and with approval from the animal care and use committee at UT Southwestern Medical Center. In vivo models were generated by subcutaneous injection of cell suspensions containing 5 × 10^6^ cells of MIA PaCa-2/MIA PaCa-2^R^ or Calu1/Calu1^R^ cells in 150-μl PBS into the flank of 6-week-old female nude mice (the Jackson Laboratory). Treatment groups for the in vivo combination studies were blinded to the investigator. Animal treatments were performed by investigators without knowing the drug information. Each drug was labeled with a number. After completing the experiment, the information of the drug was revealed. Once tumors were established (around 80 to 100 mm^3^), mice were randomized into four groups, including ones that received MRTX849 (25 mg/kg) and/or dasatinib (35 mg/kg) twice a week for 3 to 6 weeks by oral gavage. Tumor volume was measured twice weekly, and mice weight was monitored. The health of the mice was monitored every 1 to 3 days throughout the experiment, and the mice were kept on a regular 12-hour light/12-hour dark cycle with normal diet in a pathogen-free barrier facility. We did not exclude samples or animals. The sample size is similar to those generally used in the field (*n* = 5 to 10 tumor per group).

### Immunohistochemistry

Immunohistochemically staining was carried out following a standard streptavidin-biotin-peroxidase complex method. Briefly, sections were deparaffinized, and nonspecific bindings were blocked with 10% normal goat serum for 30 min. Sections were then incubated with antibodies overnight at 4°C. Antibodies included ABCC1 (Cell Signaling Technology, 14685) and PCNA (Cell Signaling Technology, 2586). After PBS washes, sections were incubated with biotinylated secondary antibodies (1:20; Vector Labs, BA-1300) and HRP avidin D (1:1000; Vector Labs, A-2004). A NovaRed substrate kit (Vector Labs, SK-4800) was used to visualize the reaction, which was followed by counterstaining with hematoxylin (Abcam, ab220365). After immunostaining, the sections were scanned at ×20 magnification at standardized settings using an EVOS imaging system (Thermo Fisher Scientific) by a single investigator who was not informed of the clinical characteristics.

### Statistical analysis

Unless specified, results are expressed as means ± SD. Experiments were repeated three times unless otherwise specified. Statistical analyses were performed using GraphPad Prism (v8, GraphPad Software), R software (v3.3.3), or RStudio (2021.09.0). The significance of the differences in the assays was analyzed by Student’s *t* test or one-way or two-way analysis of variance (ANOVA), followed by Tukey’s multiple comparisons test. Comparison of survival curves was performed using a log-rank (Mantel-Cox) test. A value of *P* < 0.05 was considered significant.

## References

[1] M. V. Milburn, L. Tong, A. M. deVos, A. Brünger, Z. Yamaizumi, S. Nishimura, S. H. Kim, Molecular switch for signal transduction: Structural differences between active and inactive forms of protooncogenic *ras* proteins. Science 247, 939–945 (1990). 2406906 10.1126/science.2406906

[2] A. K. Murugan, M. Grieco, N. Tsuchida, *RAS* mutations in human cancers: Roles in precision medicine. Semin. Cancer Biol. 59, 23–35 (2019). 31255772 10.1016/j.semcancer.2019.06.007

[3] D. Tang, G. Kroemer, R. Kang, Oncogenic KRAS blockade therapy: Renewed enthusiasm and persistent challenges. Mol. Cancer 20, 128 (2021). 34607583 10.1186/s12943-021-01422-7 PMC8489073

[4] J. M. Ostrem, U. Peters, M. L. Sos, J. A. Wells, K. M. Shokat, K-Ras(G12C) inhibitors allosterically control GTP affinity and effector interactions. Nature 503, 548–551 (2013). 24256730 10.1038/nature12796 PMC4274051

[5] A. R. Moore, S. C. Rosenberg, F. McCormick, S. Malek, RAS-targeted therapies: Is the undruggable drugged? Nat. Rev. Drug Discov. 19, 533–552 (2020). 32528145 10.1038/s41573-020-0068-6 PMC7809886

[6] M. R. Janes, J. Zhang, L. S. Li, R. Hansen, U. Peters, X. Guo, Y. Chen, A. Babbar, S. J. Firdaus, L. Darjania, J. Feng, J. H. Chen, S. Li, S. Li, Y. O. Long, C. Thach, Y. Liu, A. Zarieh, T. Ely, J. M. Kucharski, L. V. Kessler, T. Wu, K. Yu, Y. Wang, Y. Yao, X. Deng, P. P. Zarrinkar, D. Brehmer, D. Dhanak, M. V. Lorenzi, D. Hu-Lowe, M. P. Patricelli, P. Ren, Y. Liu, Targeting KRAS mutant cancers with a covalent G12C-specific inhibitor. Cell 172, 578–589.e517 (2018). 29373830 10.1016/j.cell.2018.01.006

[7] F. McCormick, Sticking it to KRAS: Covalent inhibitors enter the clinic. Cancer Cell 37, 3–4 (2020). 31951561 10.1016/j.ccell.2019.12.009 PMC7891852

[8] J. Liu, X. Song, F. Kuang, Q. Zhang, Y. Xie, R. Kang, G. Kroemer, D. Tang, NUPR1 is a critical repressor of ferroptosis. Nat. Commun. 12, 647 (2021). 33510144 10.1038/s41467-021-20904-2 PMC7843652

[9] F. Skoulidis, B. T. Li, G. K. Dy, T. J. Price, G. S. Falchook, J. Wolf, A. Italiano, M. Schuler, H. Borghaei, F. Barlesi, T. Kato, A. Curioni-Fontecedro, A. Sacher, A. Spira, S. S. Ramalingam, T. Takahashi, B. Besse, A. Anderson, A. Ang, Q. Tran, O. Mather, H. Henary, G. Ngarmchamnanrith, G. Friberg, V. Velcheti, R. Govindan, Sotorasib for lung cancers with *KRAS* p.G12C mutation. N. Engl. J. Med. 384, 2371–2381 (2021). 34096690 10.1056/NEJMoa2103695 PMC9116274

[10] H. A. Blair, Sotorasib: First approval. Drugs 81, 1573–1579 (2021). 34357500 10.1007/s40265-021-01574-2 PMC8531079

[11] J. Hallin, L. D. Engstrom, L. Hargis, A. Calinisan, R. Aranda, D. M. Briere, N. Sudhakar, V. Bowcut, B. R. Baer, J. A. Ballard, M. R. Burkard, J. B. Fell, J. P. Fischer, G. P. Vigers, Y. Xue, S. Gatto, J. Fernandez-Banet, A. Pavlicek, K. Velastagui, R. C. Chao, J. Barton, M. Pierobon, E. Baldelli, E. F. Patricoin III, D. P. Cassidy, M. A. Marx, I. I. Rybkin, M. L. Johnson, S. I. Ou, P. Lito, K. P. Papadopoulos, P. A. Janne, P. Olson, J. G. Christensen, The KRAS^G12C^ inhibitor MRTX849 provides insight toward therapeutic susceptibility of KRAS-mutant cancers in mouse models and patients. Cancer Discov. 10, 54–71 (2020). 31658955 10.1158/2159-8290.CD-19-1167 PMC6954325

[12] P. A. Jänne, G. J. Riely, S. M. Gadgeel, R. S. Heist, S. I. Ou, J. M. Pacheco, M. L. Johnson, J. K. Sabari, K. Leventakos, E. Yau, L. Bazhenova, M. V. Negrao, N. A. Pennell, J. Zhang, K. Anderes, H. Der-Torossian, T. Kheoh, K. Velastegui, X. Yan, J. G. Christensen, R. C. Chao, A. I. Spira, Adagrasib in non–small-cell lung cancer harboring a KRAS^G12C^ mutation. N. Engl. J. Med. 387, 120–131 (2022). 35658005 10.1056/NEJMoa2204619

[13] M. M. Awad, S. Liu, I. I. Rybkin, K. C. Arbour, J. Dilly, V. W. Zhu, M. L. Johnson, R. S. Heist, T. Patil, G. J. Riely, J. O. Jacobson, X. Yang, N. S. Persky, D. E. Root, K. E. Lowder, H. Feng, S. S. Zhang, K. M. Haigis, Y. P. Hung, L. M. Sholl, B. M. Wolpin, J. Wiese, J. Christiansen, J. Lee, A. B. Schrock, L. P. Lim, K. Garg, M. Li, L. D. Engstrom, L. Waters, J. D. Lawson, P. Olson, P. Lito, S. I. Ou, J. G. Christensen, P. A. Janne, A. J. Aguirre, Acquired resistance to KRAS^G12C^ inhibition in cancer. N. Engl. J. Med. 384, 2382–2393 (2021). 34161704 10.1056/NEJMoa2105281 PMC8864540

[14] Y. Zhao, Y. R. Murciano-Goroff, J. Y. Xue, A. Ang, J. Lucas, T. T. Mai, A. F. Da Cruz Paula, A. Y. Saiki, D. Mohn, P. Achanta, A. E. Sisk, K. S. Arora, R. S. Roy, D. Kim, C. Li, L. P. Lim, M. Li, A. Bahr, B. R. Loomis, E. de Stanchina, J. S. Reis-Filho, B. Weigelt, M. Berger, G. Riely, K. C. Arbour, J. R. Lipford, B. T. Li, P. Lito, Diverse alterations associated with resistance to KRAS^G12C^ inhibition. Nature 599, 679–683 (2021). 34759319 10.1038/s41586-021-04065-2 PMC8887821

[15] N. S. Akhave, A. B. Biter, D. S. Hong, Mechanisms of resistance to KRAS^G12C^-targeted therapy. Cancer Discov. 11, 1345–1352 (2021). 33820777 10.1158/2159-8290.CD-20-1616 PMC8178176

[16] N. Tanaka, J. J. Lin, C. Li, M. B. Ryan, J. Zhang, L. A. Kiedrowski, A. G. Michel, M. U. Syed, K. A. Fella, M. Sakhi, I. Baiev, D. Juric, J. F. Gainor, S. J. Klempner, J. K. Lennerz, G. Siravegna, L. Bar-Peled, A. N. Hata, R. S. Heist, R. B. Corcoran, Clinical acquired resistance to KRAS^G12C^ inhibition through a novel KRAS switch-II pocket mutation and polyclonal alterations converging on RAS-MAPK reactivation. Cancer Discov. 11, 1913–1922 (2021). 33824136 10.1158/2159-8290.CD-21-0365 PMC8338755

[17] C. Fedele, S. Li, K. W. Teng, C. J. R. Foster, D. Peng, H. Ran, P. Mita, M. J. Geer, T. Hattori, A. Koide, Y. Wang, K. H. Tang, J. Leinwand, W. Wang, B. Diskin, J. Deng, T. Chen, I. Dolgalev, U. Ozerdem, G. Miller, S. Koide, K. K. Wong, B. G. Neel, SHP2 inhibition diminishes KRASG12C cycling and promotes tumor microenvironment remodeling. J. Exp. Med. 218, (2021). 10.1084/jem.20201414 PMC7549316 33045063

[18] M. B. Ryan, F. Fece de la Cruz, S. Phat, D. T. Myers, E. Wong, H. A. Shahzade, C. B. Hong, R. B. Corcoran, Vertical pathway inhibition overcomes adaptive feedback resistance to KRAS^G12C^ inhibition. Clin. Cancer Res. 26, 1633–1643 (2020). 31776128 10.1158/1078-0432.CCR-19-3523 PMC7124991

[19] M. B. Ryan, O. Coker, A. Sorokin, K. Fella, H. Barnes, E. Wong, P. Kanikarla, F. Gao, Y. Zhang, L. Zhou, S. Kopetz, R. B. Corcoran, KRAS^G12C^-independent feedback activation of wild-type *RAS* constrains KRAS^G12C^ inhibitor efficacy. Cell Rep. 39, 110993 (2022). 35732135 10.1016/j.celrep.2022.110993 PMC9809542

[20] V. Amodio, R. Yaeger, P. Arcella, C. Cancelliere, S. Lamba, A. Lorenzato, S. Arena, M. Montone, B. Mussolin, Y. Bian, A. Whaley, M. Pinnelli, Y. R. Murciano-Goroff, E. Vakiani, N. Valeri, W. L. Liao, A. Bhalkikar, S. Thyparambil, H. Y. Zhao, E. de Stanchina, S. Marsoni, S. Siena, A. Bertotti, L. Trusolino, B. T. Li, N. Rosen, F. Di Nicolantonio, A. Bardelli, S. Misale, EGFR blockade reverts resistance to KRAS^G12C^ inhibition in colorectal cancer. Cancer Discov. 10, 1129–1139 (2020). 32430388 10.1158/2159-8290.CD-20-0187 PMC7416460

[21] H. S. Solanki, E. A. Welsh, B. Fang, V. Izumi, L. Darville, B. Stone, R. Franzese, S. Chavan, F. Kinose, D. Imbody, J. M. Koomen, U. Rix, E. B. Haura, Cell type-specific adaptive signaling responses to KRAS^G12C^ inhibition. Clin. Cancer Res. 27, 2533–2548 (2021). 33619172 10.1158/1078-0432.CCR-20-3872 PMC9940280

[22] T. J. Hagenbeek, J. R. Zbieg, M. Hafner, R. Mroue, J. A. Lacap, N. M. Sodir, C. L. Noland, S. Afghani, A. Kishore, K. P. Bhat, X. Yao, S. Schmidt, S. Clausen, M. Steffek, W. Lee, P. Beroza, S. Martin, E. Lin, R. Fong, P. Di Lello, M. H. Kubala, M. N. Yang, J. T. Lau, E. Chan, A. Arrazate, L. An, E. Levy, M. N. Lorenzo, H. J. Lee, T. H. Pham, Z. Modrusan, R. Zang, Y. C. Chen, M. Kabza, M. Ahmed, J. Li, M. T. Chang, D. Maddalo, M. Evangelista, X. Ye, J. J. Crawford, A. Dey, An allosteric pan-TEAD inhibitor blocks oncogenic YAP/TAZ signaling and overcomes KRAS G12C inhibitor resistance. Nat. Cancer 4, 812–828 (2023). 37277530 10.1038/s43018-023-00577-0 PMC10293011

[23] J. Y. Xue, Y. Zhao, J. Aronowitz, T. T. Mai, A. Vides, B. Qeriqi, D. Kim, C. Li, E. de Stanchina, L. Mazutis, D. Risso, P. Lito, Rapid non-uniform adaptation to conformation-specific KRAS(G12C) inhibition. Nature 577, 421–425 (2020). 31915379 10.1038/s41586-019-1884-x PMC7308074

[24] Y. Adachi, K. Ito, Y. Hayashi, R. Kimura, T. Z. Tan, R. Yamaguchi, H. Ebi, Epithelial-to-mesenchymal transition is a cause of both intrinsic and acquired resistance to KRAS G12C inhibitor in KRAS G12C-mutant non-small cell lung cancer. Clin. Cancer Res. 26, 5962–5973 (2020). 32900796 10.1158/1078-0432.CCR-20-2077

[25] X. Lv, X. Lu, J. Cao, Q. Luo, Y. Ding, F. Peng, A. Pataer, D. Lu, D. Han, E. Malmberg, D. W. Chan, X. Wang, S. R. Savage, S. Mao, J. Yu, F. Peng, L. Yan, H. Meng, L. Maneix, Y. Han, Y. Chen, W. Yao, E. C. Chang, A. Catic, X. Lin, G. Miles, P. Huang, Z. Sun, B. Burt, H. Wang, J. Wang, Q. C. Yao, B. Zhang, J. A. Roth, B. W. O’Malley, M. J. Ellis, M. F. Rimawi, H. Ying, X. Chen, Modulation of the proteostasis network promotes tumor resistance to oncogenic KRAS inhibitors. Science 381, eabn4180 (2023). 37676964 10.1126/science.abn4180 PMC10720158

[26] X. Song, Z. Zhou, K. Westover, Decoding the proteostasis network in resistance to KRAS inhibitors. Innovation 4, 100526 (2023).

[27] X. D. Dong, M. Zhang, C. Y. Cai, Q. X. Teng, J. Q. Wang, Y. G. Fu, Q. Cui, K. Patel, D. T. Wang, Z. S. Chen, Overexpression of ABCB1 associated with the resistance to the KRAS-G12C specific inhibitor ARS-1620 in cancer cells. Front. Pharmacol. 13, 843829 (2022). 35281897 10.3389/fphar.2022.843829 PMC8905313

[28] J. K. Sabari, V. Velcheti, K. Shimizu, M. R. Strickland, R. S. Heist, M. Singh, N. Nayyar, A. Giobbie-Hurder, S. R. Digumarthy, J. F. Gainor, A. P. Rajan, E. Nieblas-Bedolla, A. C. Burns, J. Hallin, P. Olson, J. G. Christensen, S. C. Kurz, P. K. Brastianos, H. Wakimoto, Activity of Adagrasib (MRTX849) in brain metastases: Preclinical models and clinical data from patients with KRASG12C-mutant non-small cell lung cancer. Clin. Cancer Res. 28, 3318–3328 (2022). 35404402 10.1158/1078-0432.CCR-22-0383 PMC9662862

[29] J. A. Engelman, K. Zejnullahu, T. Mitsudomi, Y. Song, C. Hyland, J. O. Park, N. Lindeman, C. M. Gale, X. Zhao, J. Christensen, T. Kosaka, A. J. Holmes, A. M. Rogers, F. Cappuzzo, T. Mok, C. Lee, B. E. Johnson, L. C. Cantley, P. A. Jänne, MET amplification leads to gefitinib resistance in lung cancer by activating ERBB3 signaling. Science 316, 1039–1043 (2007). 17463250 10.1126/science.1141478

[30] X. Liu, M. He, L. Li, X. Wang, S. Han, J. Zhao, Y. Dong, M. Ahmad, L. Li, X. Zhang, J. Huo, Y. Liu, C. Pan, C. Wang, EMT and cancer cell stemness associated with chemotherapeutic resistance in esophageal cancer. Front. Oncol. 11, 672222 (2021). 34150636 10.3389/fonc.2021.672222 PMC8209423

[31] C. M. Koch, S. F. Chiu, M. Akbarpour, A. Bharat, K. M. Ridge, E. T. Bartom, D. R. Winter, A beginner’s guide to analysis of RNA sequencing data. Am. J. Respir. Cell Mol. Biol. 59, 145–157 (2018). 29624415 10.1165/rcmb.2017-0430TR PMC6096346

[32] M. Drosten, M. Barbacid, Targeting the MAPK pathway in KRAS-driven tumors. Cancer Cell 37, 543–550 (2020). 32289276 10.1016/j.ccell.2020.03.013

[33] W. Zhang, H. T. Liu, MAPK signal pathways in the regulation of cell proliferation in mammalian cells. Cell Res. 12, 9–18 (2002). 11942415 10.1038/sj.cr.7290105

[34] E. M. Terrell, D. K. Morrison, Ras-mediated activation of the Raf family kinases. Cold Spring Harb. Perspect. Med. 9, a033746 (2019). 29358316 10.1101/cshperspect.a033746 PMC6311149

[35] M. Prieß, H. Göddeke, G. Groenhof, L. V. Schäfer, Molecular mechanism of ATP hydrolysis in an ABC transporter. ACS Cent. Sci. 4, 1334–1343 (2018). 30410971 10.1021/acscentsci.8b00369 PMC6202651

[36] P. M. Juan-Carlos, P. P. Perla-Lidia, M. M. Stephanie-Talia, A. M. Mónica-Griselda, T. E. Luz-María, ABC transporter superfamily. An updated overview, relevance in cancer multidrug resistance and perspectives with personalized medicine. Mol. Biol. Rep. 48, 1883–1901 (2021). 33616835 10.1007/s11033-021-06155-w

[37] Y. S. Tsai, M. G. Woodcock, S. H. Azam, L. B. Thorne, K. L. Kanchi, J. S. Parker, B. G. Vincent, C. V. Pecot, Rapid idiosyncratic mechanisms of clinical resistance to KRAS G12C inhibition. J. Clin. Invest. 132, e155523 (2022). 34990404 10.1172/JCI155523 PMC8843735

[38] P. Mitra, C. A. Oskeritzian, S. G. Payne, M. A. Beaven, S. Milstien, S. Spiegel, Role of ABCC1 in export of sphingosine-1-phosphate from mast cells. Proc. Natl. Acad. Sci. U.S.A. 103, 16394–16399 (2006). 17050692 10.1073/pnas.0603734103 PMC1637593

[39] M. Cargnello, P. P. Roux, Activation and function of the MAPKs and their substrates, the MAPK-activated protein kinases. Microbiol. Mol. Biol. Rev. 75, 50–83 (2011). 21372320 10.1128/MMBR.00031-10 PMC3063353

[40] C. Weiss, S. Schneider, E. F. Wagner, X. Zhang, E. Seto, D. Bohmann, JNK phosphorylation relieves HDAC3-dependent suppression of the transcriptional activity of c-Jun. EMBO J. 22, 3686–3695 (2003). 12853483 10.1093/emboj/cdg364 PMC165634

[41] P. Angel, M. Karin, The role of Jun, Fos and the AP-1 complex in cell-proliferation and transformation. Biochim. Biophys. Acta 1072, 129–157 (1991). 1751545 10.1016/0304-419x(91)90011-9

[42] M. Yoo, J. Shin, J. Kim, K. A. Ryall, K. Lee, S. Lee, M. Jeon, J. Kang, A. C. Tan, DSigDB: Drug signatures database for gene set analysis. Bioinformatics 31, 3069–3071 (2015). 25990557 10.1093/bioinformatics/btv313 PMC4668778

[43] A. Ianevski, A. K. Giri, T. Aittokallio, SynergyFinder 2.0: Visual analytics of multi-drug combination synergies. Nucleic Acids Res. 48, W488–W493 (2020). 32246720 10.1093/nar/gkaa216 PMC7319457

[44] M. Talpaz, N. P. Shah, H. Kantarjian, N. Donato, J. Nicoll, R. Paquette, J. Cortes, S. O’Brien, C. Nicaise, E. Bleickardt, M. A. Blackwood-Chirchir, V. Iyer, T. T. Chen, F. Huang, A. P. Decillis, C. L. Sawyers, Dasatinib in imatinib-resistant Philadelphia chromosome-positive leukemias. N. Engl. J. Med. 354, 2531–2541 (2006). 16775234 10.1056/NEJMoa055229

[45] J. F. Apperley, J. E. Cortes, D. W. Kim, L. Roy, G. J. Roboz, G. Rosti, E. O. Bullorsky, E. Abruzzese, A. Hochhaus, D. Heim, C. A. de Souza, R. A. Larson, J. H. Lipton, H. J. Khoury, H. J. Kim, C. Sillaber, T. P. Hughes, P. Erben, J. Van Tornout, R. M. Stone, Dasatinib in the treatment of chronic myeloid leukemia in accelerated phase after imatinib failure: The START a trial. J. Clin. Oncol. 27, 3472–3479 (2009). 19487385 10.1200/JCO.2007.14.3339 PMC4979080

[46] R. Foà, R. Bassan, A. Vitale, L. Elia, A. Piciocchi, M. C. Puzzolo, M. Canichella, P. Viero, F. Ferrara, M. Lunghi, F. Fabbiano, M. Bonifacio, N. Fracchiolla, P. Di Bartolomeo, A. Mancino, M. S. De Propris, M. Vignetti, A. Guarini, A. Rambaldi, S. Chiaretti, Dasatinib-blinatumomab for Ph-positive acute lymphoblastic leukemia in adults. N. Engl. J. Med. 383, 1613–1623 (2020). 33085860 10.1056/NEJMoa2016272

[47] P. Lito, M. Solomon, L. S. Li, R. Hansen, N. Rosen, Allele-specific inhibitors inactivate mutant KRAS G12C by a trapping mechanism. Science 351, 604–608 (2016). 26841430 10.1126/science.aad6204 PMC4955282

[48] E. C. Lerner, T. E. Smithgall, SH3-dependent stimulation of Src-family kinase autophosphorylation without tail release from the SH2 domain in vivo. Nat. Struct. Biol. 9, 365–369 (2002). 11976726 10.1038/nsb782

[49] G. Du, S. Rao, D. Gurbani, N. J. Henning, J. Jiang, J. Che, A. Yang, S. B. Ficarro, J. A. Marto, A. J. Aguirre, P. K. Sorger, K. D. Westover, T. Zhang, N. S. Gray, Structure-based design of a potent and selective covalent inhibitor for SRC kinase that targets a P-loop cysteine. J. Med. Chem. 63, 1624–1641 (2020). 31935084 10.1021/acs.jmedchem.9b01502 PMC7493195

[50] D. Gurbani, G. Du, N. J. Henning, S. Rao, A. K. Bera, T. Zhang, N. S. Gray, K. D. Westover, Structure and characterization of a covalent inhibitor of Src kinase. Front. Mol. Biosci. 7, 81 (2020). 32509799 10.3389/fmolb.2020.00081 PMC7248381

[51] D. S. Spassov, A. Ruiz-Saenz, A. Piple, M. M. Moasser, A dimerization function in the intrinsically disordered N-terminal region of Src. Cell Rep. 25, 449–463.e444 (2018). 30304684 10.1016/j.celrep.2018.09.035 PMC6226010

[52] J. B. Fell, J. P. Fischer, B. R. Baer, J. F. Blake, K. Bouhana, D. M. Briere, K. D. Brown, L. E. Burgess, A. C. Burns, M. R. Burkard, H. Chiang, M. J. Chicarelli, A. W. Cook, J. J. Gaudino, J. Hallin, L. Hanson, D. P. Hartley, E. J. Hicken, G. P. Hingorani, R. J. Hinklin, M. J. Mejia, P. Olson, J. N. Otten, S. P. Rhodes, M. E. Rodriguez, P. Savechenkov, D. J. Smith, N. Sudhakar, F. X. Sullivan, T. P. Tang, G. P. Vigers, L. Wollenberg, J. G. Christensen, M. A. Marx, Identification of the clinical development candidate MRTX849, a covalent KRAS^G12C^ inhibitor for the treatment of cancer. J. Med. Chem. 63, 6679–6693 (2020). 32250617 10.1021/acs.jmedchem.9b02052

[53] C. Hekim, M. Ilander, J. Yan, E. Michaud, R. Smykla, M. Vähä-Koskela, P. Savola, S. Tähtinen, L. Saikko, A. Hemminki, P. E. Kovanen, K. Porkka, F. Y. Lee, S. Mustjoki, Dasatinib changes immune cell profiles concomitant with reduced tumor growth in several murine solid tumor models. Cancer Immunol. Res. 5, 157–169 (2017). 28073775 10.1158/2326-6066.CIR-16-0061-T

[54] D. J. Sisler, T. K. Hinz, A. T. Le, E. K. Kleczko, R. A. Nemenoff, L. E. Heasley, Evaluation of KRAS^G12C^ inhibitor responses in novel murine KRAS^G12C^ lung cancer cell line models. Front. Oncol. 13, 1094123 (2023). 36845684 10.3389/fonc.2023.1094123 PMC9945252

[55] M. Talpaz, G. Saglio, E. Atallah, P. Rousselot, Dasatinib dose management for the treatment of chronic myeloid leukemia. Cancer 124, 1660–1672 (2018). 29370463 10.1002/cncr.31232 PMC5901015

[56] H. Wang, S. Li, Z. Cui, T. Qin, H. Shi, J. Ma, L. Li, G. Yu, T. Jiang, C. Li, Analysis of spleen histopathology, splenocyte composition and haematological parameters in four strains of mice infected with *Plasmodium berghei* K173. Malar. J. 20, 249 (2021). 34090420 10.1186/s12936-021-03786-z PMC8180108

[57] A. Mohanty, A. Nam, S. Srivastava, J. Jones, B. Lomenick, S. S. Singhal, L. Guo, H. Cho, A. Li, A. Behal, T. Mirzapoiazova, E. Massarelli, M. Koczywas, L. D. Arvanitis, T. Walser, V. Villaflor, S. Hamilton, I. Mambetsariev, M. Sattler, M. W. Nasser, M. Jain, S. K. Batra, R. Soldi, S. Sharma, M. Fakih, S. K. Mohanty, A. Mainan, X. Wu, Y. Chen, Y. He, T.-F. Chou, S. Roy, J. Orban, P. Kulkarni, R. Salgia, Acquired resistance to KRAS G12C small-molecule inhibitors via genetic/nongenetic mechanisms in lung cancer. Sci. Adv. 9, eade3816 (2023). 37831779 10.1126/sciadv.ade3816 PMC10575592

[58] M. M. Gottesman, T. Fojo, S. E. Bates, Multidrug resistance in cancer: Role of ATP-dependent transporters. Nat. Rev. Cancer 2, 48–58 (2002). 11902585 10.1038/nrc706

[59] C. J. Chen, J. E. Chin, K. Ueda, D. P. Clark, I. Pastan, M. M. Gottesman, I. B. Roninson, Internal duplication and homology with bacterial transport proteins in the *mdr1* (P-glycoprotein) gene from multidrug-resistant human cells. Cell 47, 381–389 (1986). 2876781 10.1016/0092-8674(86)90595-7

[60] S. P. Cole, G. Bhardwaj, J. H. Gerlach, J. E. Mackie, C. E. Grant, K. C. Almquist, A. J. Stewart, E. U. Kurz, A. M. Duncan, R. G. Deeley, Overexpression of a transporter gene in a multidrug-resistant human lung cancer cell line. Science 258, 1650–1654 (1992). 1360704 10.1126/science.1360704

[61] K. F. Ejendal, C. A. Hrycyna, Multidrug resistance and cancer: The role of the human ABC transporter ABCG2. Curr. Protein Pept. Sci. 3, 503–511 (2002). 12369998 10.2174/1389203023380521

[62] N. H. C. Loos, I. A. Retmana, W. Li, M. L. F. Martins, M. C. Lebre, R. W. Sparidans, J. H. Beijnen, A. H. Schinkel, ABCB1 limits brain exposure of the KRAS^G12C^ inhibitor sotorasib, whereas ABCB1, CYP3A, and possibly OATP1a/1b restrict its oral availability. Pharmacol. Res. 178, 106137 (2022). 35192958 10.1016/j.phrs.2022.106137

[63] Y. Liu, L. Wu, H. Lu, E. Wu, J. Ni, X. Zhou, Enhancing the therapeutic efficacy of KRAS^G12C^ inhibitors in lung adenocarcinoma cell models by cotargeting the MAPK pathway or HSP90. J. Oncol. 2021, 2721466 (2021). 34858498 10.1155/2021/2721466 PMC8632397

[64] Y. Zhang, C. Li, C. Xia, K. K. W. To, Z. Guo, C. Ren, L. Wen, F. Wang, L. Fu, N. Liao, Adagrasib, a KRAS G12C inhibitor, reverses the multidrug resistance mediated by ABCB1 in vitro and in vivo. Cell Commun. Signal 20, 142 (2022). 36104708 10.1186/s12964-022-00955-8 PMC9472360

[65] M. Müller, C. Meijer, G. J. Zaman, P. Borst, R. J. Scheper, N. H. Mulder, E. G. de Vries, P. L. Jansen, Overexpression of the gene encoding the multidrug resistance-associated protein results in increased ATP-dependent glutathione S-conjugate transport. Proc. Natl. Acad. Sci. U.S.A. 91, 13033–13037 (1994). 7809167 10.1073/pnas.91.26.13033 PMC45575

[66] G. Conseil, S. P. Cole, Two polymorphic variants of ABCC1 selectively alter drug resistance and inhibitor sensitivity of the multidrug and organic anion transporter multidrug resistance protein 1. Drug Metab. Dispos. 41, 2187–2196 (2013). 24080162 10.1124/dmd.113.054213

[67] G. Nadel, Z. Yao, I. Ben-Ami, Z. Naor, R. Seger, Gq-induced apoptosis is mediated by AKT inhibition that leads to PKC-induced JNK activation. Cell. Physiol. Biochem. 50, 121–135 (2018). 30278445 10.1159/000493963

[68] B. Zhang, Y. Zhang, J. Zhang, P. Liu, B. Jiao, Z. Wang, R. Ren, Focal adhesion kinase (FAK) inhibition synergizes with KRAS G12C inhibitors in treating cancer through the regulation of the FAK-YAP signaling. Adv. Sci. 8, e2100250 (2021). 10.1002/advs.202100250 PMC8373085 34151545

[69] K. M. Hanssen, M. Haber, J. I. Fletcher, Targeting multidrug resistance-associated protein 1 (MRP1)-expressing cancers: Beyond pharmacological inhibition. Drug Resist. Updat. 59, 100795 (2021). 34983733 10.1016/j.drup.2021.100795

[70] L. C. Kim, L. Song, E. B. Haura, Src kinases as therapeutic targets for cancer. Nat. Rev. Clin. Oncol. 6, 587–595 (2009). 19787002 10.1038/nrclinonc.2009.129

[71] G. Saturno, F. Lopes, I. Niculescu-Duvaz, D. Niculescu-Duvaz, A. Zambon, L. Davies, L. Johnson, N. Preece, R. Lee, A. Viros, D. Holovanchuk, M. Pedersen, R. McLeary, P. Lorigan, N. Dhomen, C. Fisher, U. Banerji, E. Dean, M. G. Krebs, M. Gore, J. Larkin, R. Marais, C. Springer, The paradox-breaking panRAF plus SRC family kinase inhibitor, CCT3833, is effective in mutant KRAS-driven cancers. Ann. Oncol. 32, 269–278 (2021). 33130216 10.1016/j.annonc.2020.10.483 PMC7839839

[72] J. Araujo, C. Logothetis, Dasatinib: A potent SRC inhibitor in clinical development for the treatment of solid tumors. Cancer Treat. Rev. 36, 492–500 (2010). 20226597 10.1016/j.ctrv.2010.02.015 PMC3940067

[73] D. S. Tan, B. Haaland, J. M. Gan, S. C. Tham, I. Sinha, E. H. Tan, K. H. Lim, A. Takano, S. S. Krisna, M. M. Thu, H. P. Liew, A. Ullrich, W. T. Lim, B. T. Chua, Bosutinib inhibits migration and invasion via ACK1 in KRAS mutant non-small cell lung cancer. Mol. Cancer 13, 13 (2014). 24461128 10.1186/1476-4598-13-13 PMC3930897

[74] A. J. de Langen, M. L. Johnson, J. Mazieres, A. C. Dingemans, G. Mountzios, M. Pless, J. Wolf, M. Schuler, H. Lena, F. Skoulidis, Y. Yoneshima, S. W. Kim, H. Linardou, S. Novello, A. J. van der Wekken, Y. Chen, S. Peters, E. Felip, B. J. Solomon, S. S. Ramalingam, C. Dooms, C. R. Lindsay, C. G. Ferreira, N. Blais, C. C. Obiozor, Y. Wang, B. Mehta, T. Varrieur, G. Ngarmchamnanrith, B. Stollenwerk, D. Waterhouse, L. Paz-Ares, Sotorasib versus docetaxel for previously treated non-small-cell lung cancer with KRAS^G12C^ mutation: A randomised, open-label, phase 3 trial. Lancet 401, 733–746 (2023). 36764316 10.1016/S0140-6736(23)00221-0

[75] E. L. Deer, J. González-Hernández, J. D. Coursen, J. E. Shea, J. Ngatia, C. L. Scaife, M. A. Firpo, S. J. Mulvihill, Phenotype and genotype of pancreatic cancer cell lines. Pancreas 39, 425–435 (2010). 20418756 10.1097/MPA.0b013e3181c15963 PMC2860631

[76] E. A. McMillan, M. J. Ryu, C. H. Diep, S. Mendiratta, J. R. Clemenceau, R. M. Vaden, J. H. Kim, T. Motoyaji, K. R. Covington, M. Peyton, K. Huffman, X. Wu, L. Girard, Y. Sung, P. H. Chen, P. L. Mallipeddi, J. Y. Lee, J. Hanson, S. Voruganti, Y. Yu, S. Park, J. Sudderth, C. DeSevo, D. M. Muzny, H. Doddapaneni, A. Gazdar, R. A. Gibbs, T. H. Hwang, J. V. Heymach, I. Wistuba, K. R. Coombes, N. S. Williams, D. A. Wheeler, J. B. MacMillan, R. J. Deberardinis, M. G. Roth, B. A. Posner, J. D. Minna, H. S. Kim, M. A. White, Chemistry-first approach for nomination of personalized treatment in lung cancer. Cell 173, 864–878.e29 (2018). 29681454 10.1016/j.cell.2018.03.028 PMC5935540

[77] E. B. Krall, B. Wang, D. M. Munoz, N. Ilic, S. Raghavan, M. J. Niederst, K. Yu, D. A. Ruddy, A. J. Aguirre, J. W. Kim, A. J. Redig, J. F. Gainor, J. A. Williams, J. M. Asara, J. G. Doench, P. A. Janne, A. T. Shaw, R. E. McDonald III, J. A. Engelman, F. Stegmeier, M. R. Schlabach, W. C. Hahn, KEAP1 loss modulates sensitivity to kinase targeted therapy in lung cancer. eLife 6, e18970 (2017). 28145866 10.7554/eLife.18970 PMC5305212

[78] C. Y. Wu, Y. Feng, E. R. Cardenas, N. Williams, P. E. Floreancig, J. K. De Brabander, M. G. Roth, Studies toward the unique pederin family member psymberin: Structure-activity relationships, biochemical studies, and genetics identify the mode-of-action of psymberin. J. Am. Chem. Soc. 134, 18998–19003 (2012). 23088155 10.1021/ja3057002 PMC3504174

[79] N. Fedchenko, J. Reifenrath, Different approaches for interpretation and reporting of immunohistochemistry analysis results in the bone tissue—A review. Diagn. Pathol. 9, 221 (2014). 25432701 10.1186/s13000-014-0221-9 PMC4260254

