# Supplementary material for: SRC kinase drives multidrug resistance induced by KRAS-G12C inhibition
Source: Sci Adv. 2024 Dec 11;10(50):eadq4274. doi: 10.1126/sciadv.adq4274 (PMC11633746; doi:10.1126/sciadv.adq4274)
Supplement: Supplementary file 1 — Figs. S1 to S6 [file sciadv.adq4274_sm.pdf]

Science Advances
AAAS

# Supplementary Materials for

## SRC kinase drives multidrug resistance induced by KRAS-G12C inhibition

Xinxin Song *et al.*

Corresponding author: Xinxin Song, xinxin.song@utsouthwestern.edu;
Daolin Tang, daolin.tang@utsouthwestern.edu

*Sci. Adv.* **10**, eadq4274 (2024)
DOI: 10.1126/sciadv.adq4274

**This PDF file includes:**

Figs. S1 to S6

# Fig.S1

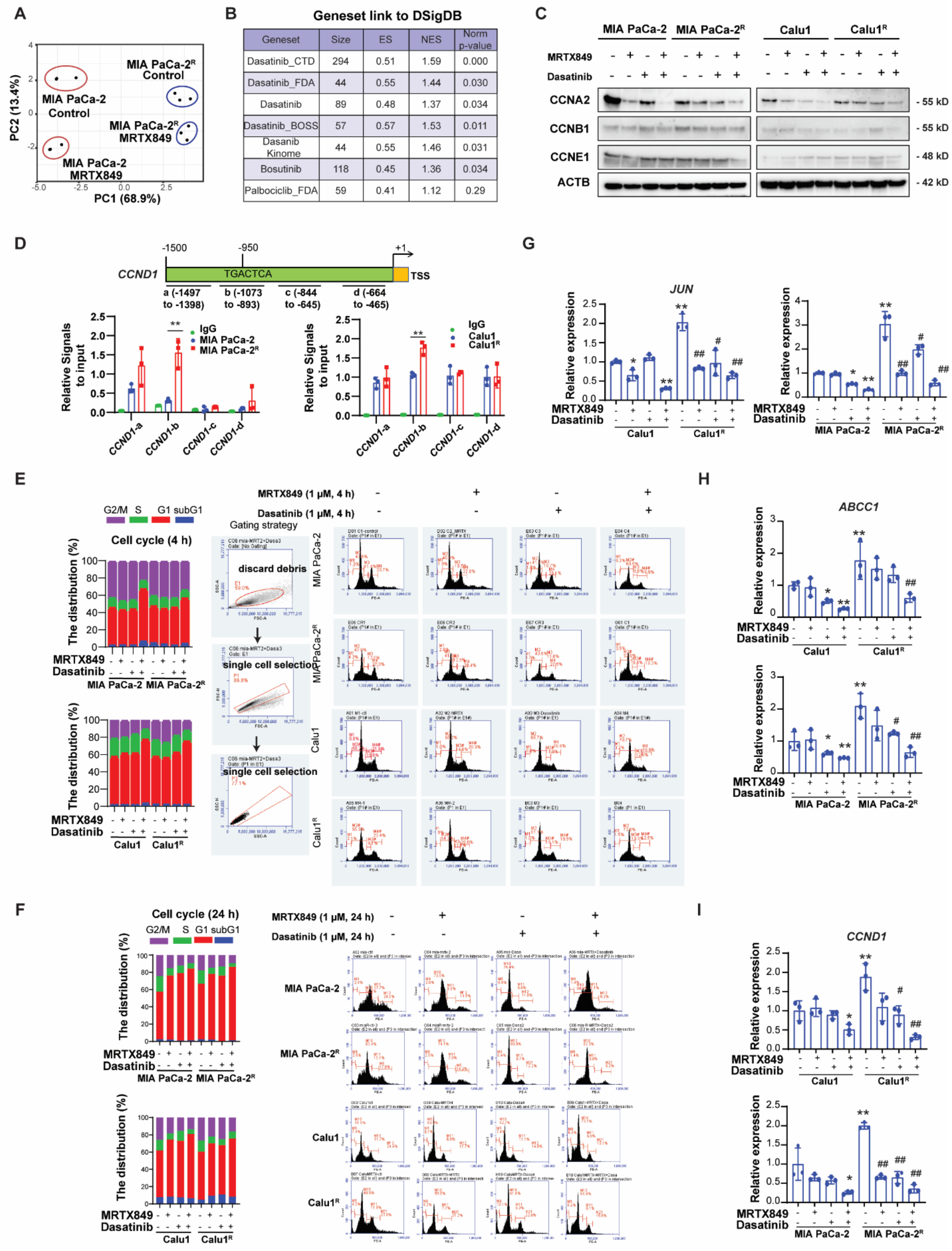


**B** Geneset link to DSigDB

| Geneset | Size | ES | NES | Norm p-value |
|---|---|---|---|---|
| Dasatinib_CTD | 294 | 0.51 | 1.59 | 0.000 |
| Dasatinib_FDA | 44 | 0.55 | 1.44 | 0.030 |
| Dasatinib | 89 | 0.48 | 1.37 | 0.034 |
| Dasatinib_BOSS | 57 | 0.57 | 1.53 | 0.011 |
| Dasanib Kinome | 44 | 0.55 | 1.46 | 0.031 |
| Bosutinib | 118 | 0.45 | 1.36 | 0.034 |
| Palbociclib_FDA | 59 | 0.41 | 1.12 | 0.29 |

**Figure S1. Screening of FDA-approved drugs identifies that dasatinib enhances the efficacy of MRTX849.**

(A) Principal component analysis of next-generation sequencing of vehicle versus MRTX849 treatment in MIA PaCa-2 and MIA PaCa-$2^R$ cells. Unit variance scaling is applied to rows; singular value decomposition with imputation was used to calculate principal components. X and Y axes show principal component 1 and principal component 2, which explain 68.9% and 13.4% of the total variance, respectively. Prediction ellipses are such that with a probability of 0.95, a new observation from the same group will fall inside the ellipse.

(B) Differential gene expression RNA-seq data of the resistant cells versus parental cells together with DSigDB (*67*) suggests the candidate drugs as possible therapeutic options for MRTX849-resistant cells.

(C) Immunoblot analysis of the indicated proteins of cells treated with 1 μM dasatinib and 1 μM MRTX849 for 24 h. The blots shown are representative of three repeats.

(D) The *CCND1* promoter region is divided into a, b, c, and d fractions. Chromatin immunoprecipitation assays were performed using digested chromatin from the indicated cells and anti-JUN antibodies. The binding DNA was purified. The *CCND1* promoter fractions a-d were measured by quantitative real-time PCR (n = 3 biologically independent samples; $^{**}p < 0.01$; two-way ANOVA; data are presented as mean ± SD).

(E) Cell cycle analysis of the combination of MRTX849 (1 μM, 4 h) and dasatinib (1 μM, 4 h) in MIA PaCa-2/MIA PaCa-$2^R$ and Calu1/Calu$1^R$ cells. Representative gating strategy and flow cytometry plots showing the cell cycle analysis of the combination of MRTX849 and dasatinib in MIA PaCa-2/MIA PaCa-$2^R$ and Calu1/Calu$1^R$ cells.

(F) Cell cycle analysis of the combination of MRTX849 (1 μM, 24 h) and dasatinib (1 μM, 4 or 24 h) in MIA PaCa-2/MIA PaCa-$2^R$ and Calu1/Calu$1^R$ cells. Representative flow cytometry plots showing the cell cycle analysis of the combination of MRTX849 (1 μM, 24 h) and dasatinib (1 μM, 24 h).

(G-I) Cells were treated with 1 μM dasatinib and 1 μM MRTX849 for 24 h followed by quantitative real-time PCR to measure the expression of JUN (G), ABCC1 (H) and CCND1 (I) in MIA PaCa-2/MIA PaCa-$2^R$ and Calu1/Calu$1^R$ cells (n = 3 biologically independent samples; *p < 0.05 or **p < 0.01 compared to gene expression in the parental cells; #p < 0.05 or ##p < 0.01 compared to gene expression in the resistant cells; two-way ANOVA with Tukey's multiple comparisons test; data are presented as mean ± SD).

Fig.S2

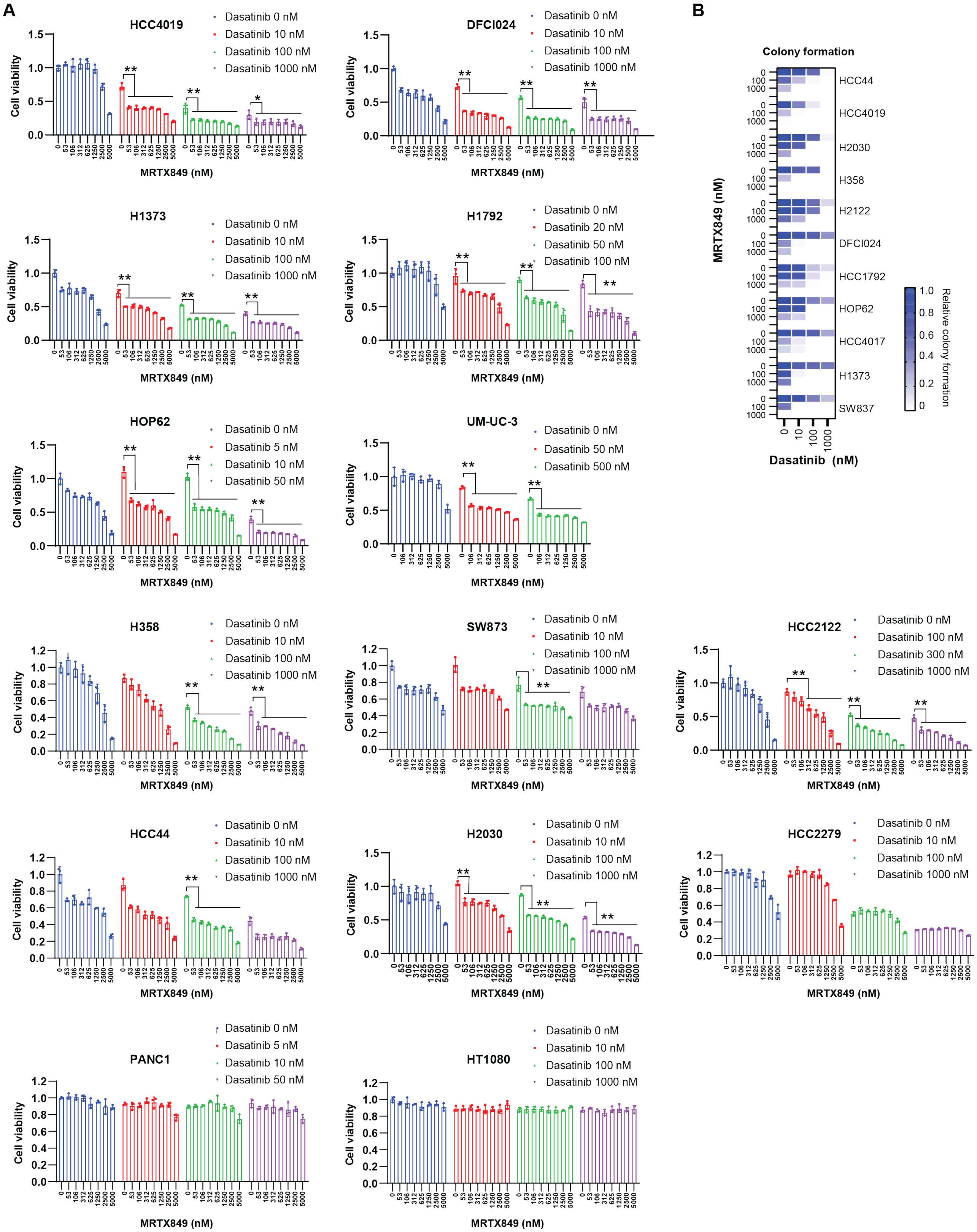
A
B
HCC4019
DFCI024
H1373
H1792
HOP62
UM-UC-3
H358
SW873
HCC2122
HCC44
H2030
HCC2279
PANC1
HT1080
Dasatinib 0 nM
Dasatinib 5 nM
Dasatinib 10 nM
Dasatinib 20 nM
Dasatinib 50 nM
Dasatinib 100 nM
Dasatinib 300 nM
Dasatinib 500 nM
Dasatinib 1000 nM
Cell viability
MRTX849 (nM)
Colony formation
HCC44
HCC4019
H2030
H358
H2122
DFCI024
HCC1792
HOP62
HCC4017
H1373
SW837
MRTX849 (nM)
Dasatinib (nM)
Relative colony formation

**Figure S2. Cell viability and colony formation after the combination of MRTX849 and dasatinib in multiple KRAS-G12C cell lines.**
(A) *KRAS-G12C*–mutant cell lines, including SW873, H358, HCC44, HCC4019, DFCI024, HCC2122, H1373, H1792, H2030, HOP62 and UM-UC-3 cells, as well as the *G12D*-mutant PANC1 and KRAS WT cell lines, including HT1080 and HCC2279, were treated with the indicated doses of MRTX849 and dasatinib for 3 days. Relative cell viability was measured by CCK8 assay (n = 3 biologically independent samples; $*p < 0.05$, $**p < 0.01$; two-way ANOVA with Tukey's multiple comparisons test; data are presented as mean ± SD).
(B) Cell clonogenic assay. *KRAS-G12C*–mutant cell lines, including HCC44, HCC4019, H2030, H358, H2122, DFCI024, HCC1792, HOP62, HCC4017, HCC1373, and SW837, were treated with MRTX849 (0, 100, or 1000 nM) and dasatinib (0, 0.2, 1, or 5 μM) for 24 h, observed for 10-20 days and then crystal violet staining was evaluated. Heatmap of relative colony formation is shown.

# Fig.S3

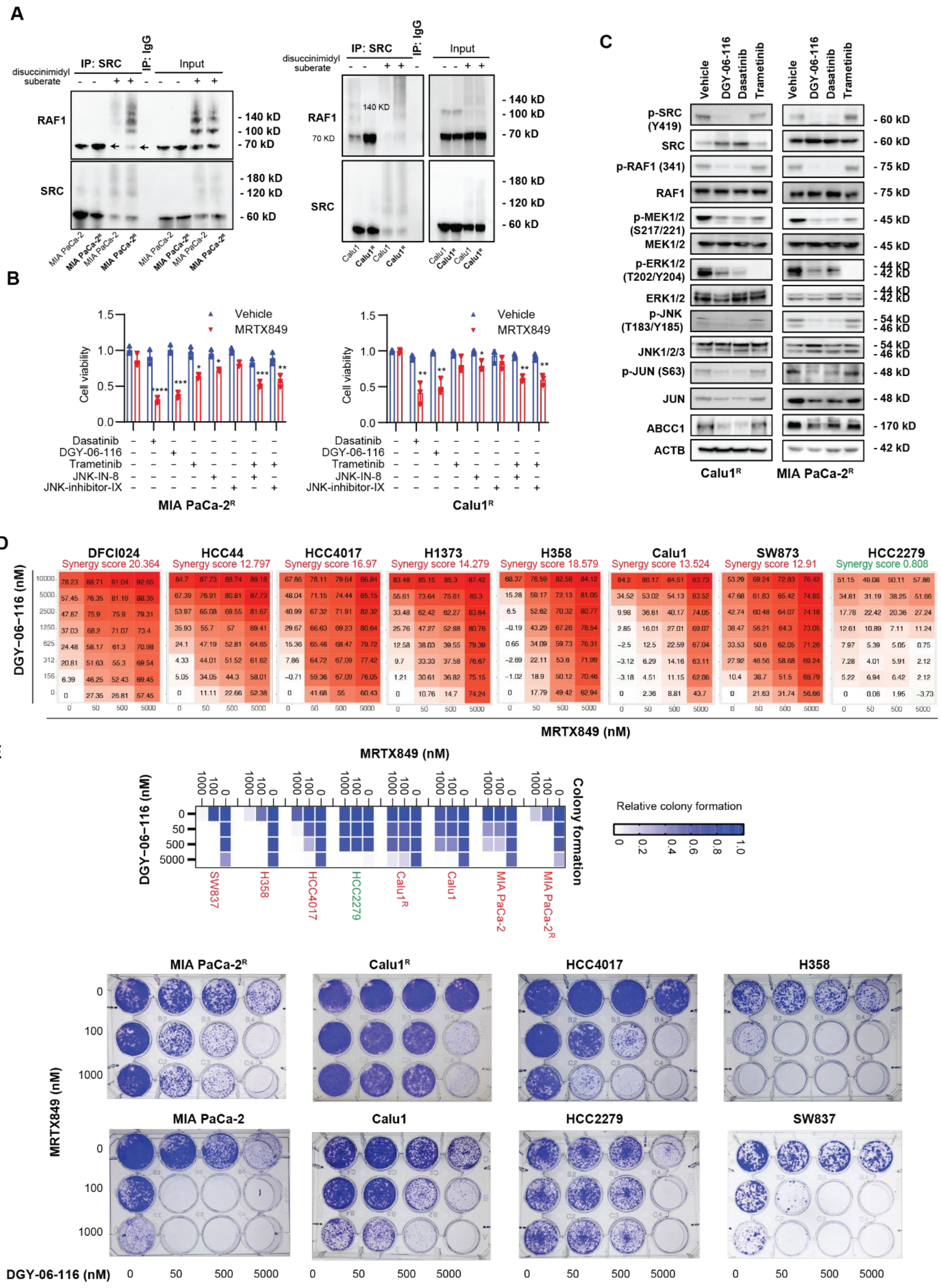

A
IP: SRC
IP: IgG
Input
disuccinimidyl suberate
RAF1
SRC
- 140 kD
- 100 kD
- 70 kD
- 180 kD
- 120 kD
- 60 kD
MIA PaCa-2
MIA PaCa-2R
Calu1
Calu1R
B
Vehicle
MRTX849
Cell viability
Dasatinib
DGY-06-116
Trametinib
JNK-IN-8
JNK-inhibitor-IX
MIA PaCa-2R
Calu1R
C
Vehicle
DGY-06-116
Dasatinib
Trametinib
p-SRC (Y419)
SRC
p-RAF1 (341)
RAF1
p-MEK1/2 (S217/221)
MEK1/2
p-ERK1/2 (T202/Y204)
ERK1/2
p-JNK (T183/Y185)
JNK1/2/3
p-JUN (S63)
JUN
ABCC1
ACTB
- 60 kD
- 75 kD
- 45 kD
- 44 kD
- 42 kD
- 54 kD
- 46 kD
- 48 kD
- 170 kD
- 42 kD
Calu1R
MIA PaCa-2R
D
DFCI024
Synergy score 20.364
HCC44
Synergy score 12.797
HCC4017
Synergy score 16.97
H1373
Synergy score 14.279
H358
Synergy score 18.579
Calu1
Synergy score 13.524
SW873
Synergy score 12.91
HCC2279
Synergy score 0.808
DGY-06-116 (nM)
MRTX849 (nM)
E
MRTX849 (nM)
DGY-06-116 (nM)
Colony formation
Relative colony formation
SW837
H358
HCC4017
HCC2279
Calu1R
Calu1
MIA PaCa-2
MIA PaCa-2R
F
MIA PaCa-2R
Calu1R
HCC4017
H358
MIA PaCa-2
Calu1
HCC2279
SW837
MRTX849 (nM)
DGY-06-116 (nM)

**Figure S3. SRC positively regulates MRTX849-induced resistance.**
**(A)** Cells were treated in the absence or presence of 1 mM disuccinimidyl suberate (DSS), and then immunoprecipitation assays were performed with anti-SRC antibodies followed by western blotting. RAF1 dimerization was increasingly binding with SRC in the resistant cells. The blots shown are representative of three repeats.
**(B)** MIA PaCa-2$^R$ and Calu1$^R$ cells were treated with the SRC inhibitors (dasatinib [1 μM] or DGY-06-116 [1 μM]), MEK inhibitor (trametinib; 1 μM), and JNK inhibitors (JNK inhibitor IX [0.1 μM] or JNK-IN-8 [1 μM]) in the absence or presence of 1 μM of MRTX849 for 72 h and then cell viability was assayed (n = 3 biologically independent samples; *$p < 0.05$, **$p < 0.01$, ***$p < 0.001$, or ****$p < 0.0001$ compared to cell viability in the vehicle group treated with MRTX849; two-way ANOVA with Tukey's multiple comparisons test).
**(C)** Cells were treated with 1 μM of MRTX849, dasatinib, DGY-06-116, or trametinib for 24 h followed by immunoblot analysis. The blots shown are representative of three repeats.
**(D)** *KRAS-G12C*–mutant cell lines, including DFCI024, HCC44, HCC4017, H1373, H358, Calu1, and SW837 as well as KRAS WT cell line HCC2279 were treated with the indicated doses of MRTX849 and DGY-06-116 for 3 days, and relative cell viability was assessed. Shown is the calculation and visualization of ZIP synergy scores for the drug combination of MRTX849 and DGY-06-116 performed by SynergyFinder. The growth inhibition (%) in the dose response matrix of the indicated cell lines is shown.
**(E, F)** Cell clonogenic assay. *KRAS-G12C*–mutant cells (MIA PaCa-2, MIA PaCa-2$^R$, Calu1, Calu1$^R$, HCC4017, H358, and SW837) and KRAS WT cells (HCC2279) were treated with MRTX849 (0, 100, or 1000 nM) and DGY-06-116 (0, 0.05, 0.5, or 5 μM) for 24 h, observed for 10-20 days, and then crystal violet staining was evaluated. Photo and heatmap of relative colony formation is shown.

Fig.S4

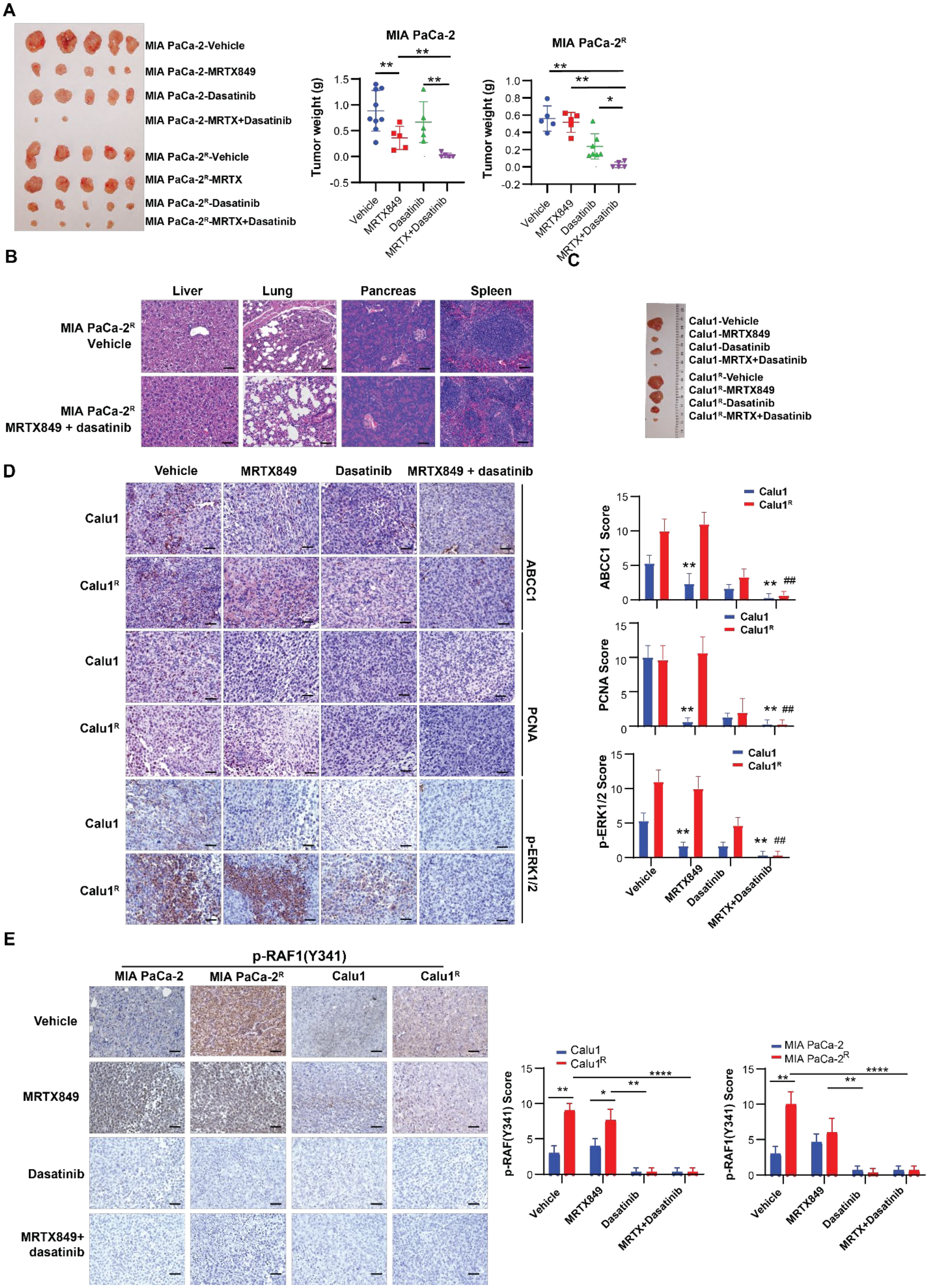
A
MIA PaCa-2-Vehicle
MIA PaCa-2-MRTX849
MIA PaCa-2-Dasatinib
MIA PaCa-2-MRTX+Dasatinib
MIA PaCa-2R-Vehicle
MIA PaCa-2R-MRTX
MIA PaCa-2R-Dasatinib
MIA PaCa-2R-MRTX+Dasatinib
MIA PaCa-2
MIA PaCa-2R
Tumor weight (g)
Vehicle
MRTX849
Dasatinib
MRTX+Dasatinib
B
Liver
Lung
Pancreas
Spleen
MIA PaCa-2R Vehicle
MIA PaCa-2R MRTX849 + dasatinib
C
Calu1-Vehicle
Calu1-MRTX849
Calu1-Dasatinib
Calu1-MRTX+Dasatinib
Calu1R-Vehicle
Calu1R-MRTX849
Calu1R-Dasatinib
Calu1R-MRTX+Dasatinib
D
Vehicle
MRTX849
Dasatinib
MRTX849 + dasatinib
Calu1
Calu1R
ABCC1
PCNA
p-ERK1/2
ABCC1 Score
PCNA Score
p-ERK1/2 Score
E
p-RAF1(Y341)
MIA PaCa-2
MIA PaCa-2R
Calu1
Calu1R
Vehicle
MRTX849
Dasatinib
MRTX849+ dasatinib
p-RAF1(Y341) Score
MRTX+Dasatinib

**Figure S4. The combination of MRTX849 and dasatinib achieved synergistic anticancer effects *in vivo*.**

**(A)** Photos of tumors on day 29. Tumor weight on day 29 (n=5; *$p < 0.05$, **$p < 0.01$, two-way ANOVA with Tukey's multiple comparisons test; data are presented as mean ± SD).

**(B)** Representative images of H&E staining of liver, lung, pancreas, and spleen in the vehicle and the combination of MRTX849 and dasatinib in MIA PaCa-2$^R$ xenograft models. Scale = 50 μm.

**(C)** Photo of tumors on day 64.

**(D)** The staining of ABCC1, p-ERK1/2, and PCNA in Calu1/Calu1$^R$ xenograft models at week 9. Scale = 50 μm. Quantification of immunohistochemistry analysis was performed by calculation using the immunoreactive score in Calu1/Calu1$^R$ xenograft models at week 9 (n = 3, *$p < 0.05$ or ** $p < 0.01$ compared with the vehicle; ##$p < 0.01$ compared with MRTX849 treatment; two-way ANOVA; data are presented as mean ± SD).

**(E)** The staining of p-RAF1 (Y341) in the xenograft models. Scale = 50 μm. Quantification of immunohistochemistry analysis was performed by calculation using the immunoreactive score (n = 3; two-way ANOVA; *$p < 0.05$, **$p < 0.01$, ****$p < 0.0001$; data are presented as mean ± SD).

# Fig.S5

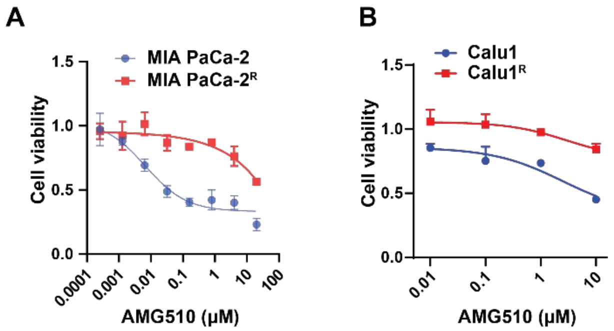


**Figure S5. MRTX849-resistant KRAS-G12C–mutant cells are resistant to AMG510.**
**(A)** MIA PaCa-2 and its resistant cells (MIA PaCa-2$^{R}$) were treated with AMG510 at the indicated concentrations. The percentage of viable cells is shown relative to untreated controls by CCK8 assay (n = 3 biologically independent samples; data are presented as mean ± SD).
**(B)** Calu1 and its KRAS-G12Ci–resistant cells (Calu1$^{R}$) were treated with AMG510 at the indicated concentrations. The percentage of viable cells is shown relative to untreated controls by CCK8 assay (n = 3 biologically independent samples; data are presented as mean ± SD).

# Fig.S6

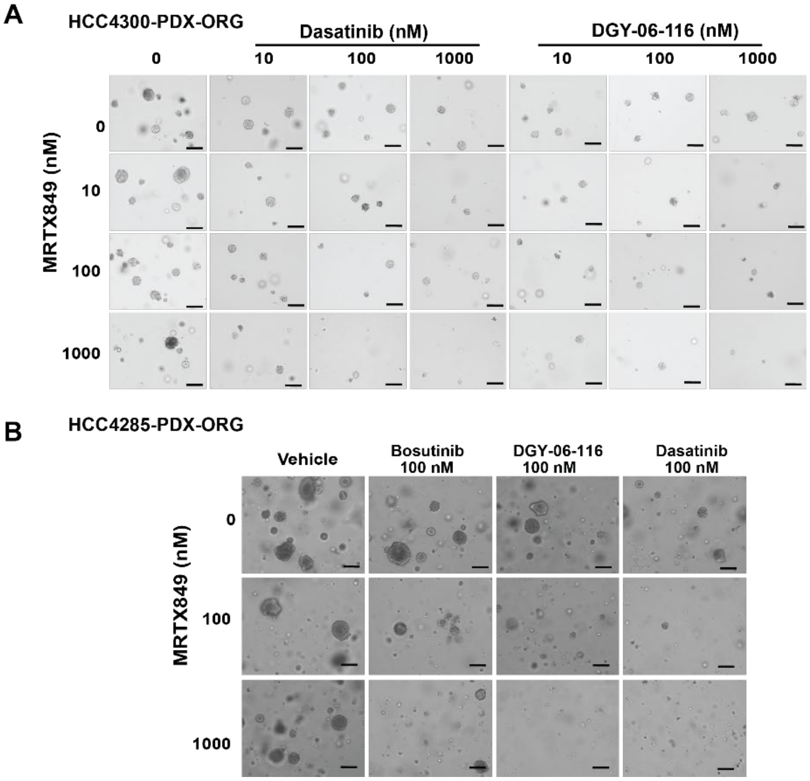


**Figure S6. Effects of MRTX849 and SRC inhibitors in the treatment of KRAS-G12C–mutant patient-derived organoids.**

**(A)** Brightfield microscopy images of patient-derived organoids HCC4300-PDX-ORG treated with vehicle, MRTX849, dasatinib, or DGY-06-116, as well as combinations, after 7 days of treatment. Scale = 500 µm.

**(B)** Brightfield microscopy images of patient-derived organoids HCC4285-PDX-ORG treated with vehicle, MRTX849, bosutinib, DGY-06-116, or dasatinib, as well as combinations, after 7 days of treatment. Scale = 300 µm.
